# Navigating entanglement via Ruderman–Kittel–Kasuya–Yosida exchange: oscillatory, boundary-residing, pulsed, and damping-stabilized trajectories

**DOI:** 10.1038/s41598-026-47292-1

**Published:** 2026-04-10

**Authors:** Son-Hsien Chen, Seng Ghee Tan, Ching-Ray Chang

**Affiliations:** 1 Department of Applied Physics and Chemistry, University of Taipei, Taipei, 100234 Taiwan; 2 Department of Optoelectric Physics, Chinese Culture University, Taipei, 11114 Taiwan; 3 Quantum Information Center, Chung Yuan Christian University, Taoyuan, 320314 Taiwan

**Keywords:** Optics and photonics, Physics

## Abstract

Entanglement dynamics are fundamental to quantum technologies, yet controlling their temporal evolution in a reversible and stable manner remains challenging. We propose a solid-state framework based on the Ruderman–Kittel–Kasuya–Yosida interaction, realizable in gate-defined quantum dots or suspended structures, in which two spin qubits couple to a central spin qudit that mediates an effective, time-dependent exchange. The dynamics are governed by an exchange-time integral that unifies interaction strength and physical time into a single scalar control variable, enabling *time-reversible* and cyclic navigation of the Hilbert space. Crucially, we show that out-of-phase modulation grants access to higher entanglement subspaces, while introducing damping to the exchange modulation achieves stabilized trajectories that drive the system toward stationary entanglement values. This framework provides a systematic route for shaping entanglement dynamics, particularly in the *near-boundary regime*, using exchange control alone, overcoming the limitations of monotonic evolution and offering practical strategies for entanglement stabilization in realistic solid-state architectures, with direct relevance to quantum metrology and environment-assisted entanglement engineering.

## Introduction

Quantum entanglement refers to the nonclassical correlations between subsystems, where the overall quantum state cannot be factored into independent states of the individual parts^1,2^. This correlation plays a pivotal role in emerging quantum technologies^3^, underpinning advances in gravitational wave detection^4,5^, quantum cryptography^6–9^, and quantum computation^2,10–15^. Despite its foundational importance, entanglement generation and the control of its dynamics remain central challenges in quantum information science^16–19^.

To address these challenges, diverse quantum processor platforms have been developed, including superconducting circuits^20^, trapped ions^21,22^, and photonic qubits^23^. Among these, spin qubits in solid-state systems are particularly promising candidates, specifically those based on magnetic impurities or defect centers (e.g., nitrogen-vacancy centers in diamond, donor spins in silicon) and lithographically or gate-defined quantum dots (QDs). Defect-based qubits combine optical initialization and readout with long coherence times, enabling remote entanglement over distances of up to two meters^24^. Donor spins in silicon achieve coherence times on the order of seconds with gate fidelities above 99%^25–27^, whereas QD spin qubits support dense integration and fast all-electrical control, with resonant CNOT gate fidelities above 98%^28–31^. Both architectures allow electrically tunable exchange coupling for rapid two-qubit gates and coherence protection via dynamical decoupling^30,32^. This gate-voltage tunability enables adjustment of the final entanglement over a broad range of strengths^33,34^.

However, realizing a scalable architecture with these spin qubits requires a robust long-range coupling mechanism, as direct exchange interaction is limited to nearest neighbors. To this end, both QDs^35–41^ and magnetic impurities^42–45^ can couple via Ruderman–Kittel–Kasuya–Yosida (RKKY) exchange^46–48^. This interaction is mediated by the host electrons, alternating between ferromagnetic and antiferromagnetic regimes with distance, which facilitates entanglement generation^43,49^, even at long range^37,50^.

Environment-mediated entanglement^51^, such as that induced by RKKY, exhibits distinctive temporal behavior. It can change abruptly, leading to entanglement sudden death (ESD)^52–56^—a complete loss of entanglement in finite time—and its counterpart, entanglement sudden birth (ESB)^33,57,58^. These phenomena, observed in various solid-state^33,58,59^ and optical systems^60^, involve ESD-ESB transitions that can be of finite (TFD) or zero duration (TZD). The parity of the mediator^61,62^ critically determines the resulting entanglement. Furthermore, because entanglement reduces spin purity^63^, accurately modeling these systems requires a fully quantum treatment, as semiclassical approaches (like the Landau-Lifshitz-Gilbert equation) fail to capture the non-conservation of local spin magnitude^64,65^.

Enhancing the tunability of entanglement often involves enabling qubit motion. Moving qubits have been explored in cavity quantum electrodynamics systems, where atoms coupled to cavity photons preserve entanglement by tuning their velocities^66–68^ or prevent ESD by manipulating cavity-cavity interactions^69^. In the solid state, strong entanglement can be created by scattering ballistic electrons off magnetic impurities^70,71^. However, a systematic and programmable method for shaping the temporal entanglement profile—or trajectory—in solid-state systems is still lacking. Resolving this is of fundamental and practical significance for sustained entanglement distribution and the exploration of the dynamically accessible Hilbert space.

In this paper, we present an RKKY-exchange-based device for the systematic shaping of entanglement trajectories. The spin qubits considered here can be implemented using suspended impurity/defect centers or stationary QD-confined spins coupled effectively through the mediating two-dimensional electron gas (2DEG). We show that the dynamics are parameterized by the exchange-time integral (ETI), which acts as an effective evolution variable and enables the reversal of previously visited states along a trajectory. In our implementation, the required time dependence of the sign change in the exchange coupling is achieved by a motion-driven scheme (prescribing the spatial vibrational motion of the qubits). Alternatively, a gate-driven scheme, which switches the exchange between ferromagnetic and antiferromagnetic regimes via dynamical voltages $V_{G}\left( t\right)$^44,72,73^, can realize the same exchange dynamics without motion. We demonstrate that distinct trajectories can be achieved by employing the boundary-proximal initial states (ISs) identified through our recent work in Ref.^59^, which are valuable for quantum metrology due to their sensitivity to external fields^74^. Furthermore, introducing damping to the out-of-phase vibrations facilitates the generation and stabilization of large entanglement. The proposed device enables reversible access to quantum states and supports applications exploiting near-boundary physics, characterized by weak entanglement. Moreover, by alternating the exchange polarity, our platform also offers an echo-like correction mechanism^32,75^, unwinding phase accumulation to mitigate dephasing.

The paper is organized as follows. Section "Model and formalism" introduces the model, describes the device, and outlines the ISs required to realize ESD, ESB, and TFD. The subsequent numerical analysis is divided into two distinguishable dynamic regimes based on the boundedness of the exchange-time-integral (ETI). Section "In-phase and antiphase vibrations" demonstrates cyclic navigation via in-phase and antiphase vibrations, where the system periodically retraces its path. In contrast, Section "Out-of-phase vibrations" examines stabilized navigation, showing how out-of-phase modulation accesses higher entanglement subspaces while damping locks the system to a stationary entanglement value. Finally, Section "Conclusions" summarizes our findings.

## Model and formalism

In this section, we delineate the device architecture, establish the theoretical framework for the system, and introduce the ETI as the governing control variable. We then define the specific boundary-proximal ISs used to investigate the entanglement dynamics.

### Hamiltonian and dynamics

**Figure 1:**
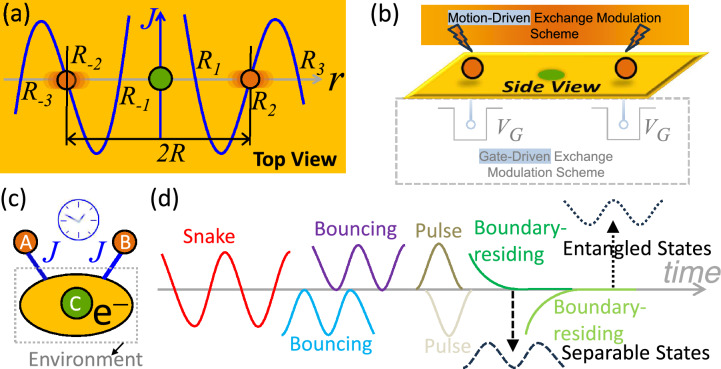
System schematic and entanglement dynamics. **a** Top view of suspended vibrating spin qubits *A* and *B* (orange) coupled via an effective, alternating RKKY exchange *J* to a central spin qudit *C* (green). This coupling is mediated by the interaction between the qubits and the local electron spin polarization induced by the central qudit. The environment comprises *C* and a two-dimensional electron gas *e* (2DEG, yellow), and the spatial RKKY profile (blue) defines the exchange nodes $R_{n}$. **b** Side view illustrating the motion-driven exchange modulation scheme. Here, the exchange coupling to the underlying local electron spins is modulated by the distance between the qubits and the *e*-spins (non-contact proximity). Alternatively, a gate-driven scheme (gray dashed box), using an applied dynamical voltage $V_{G}=V_{G}(t)$, can generate an equivalent sign-alternating exchange interaction; in this scheme, stationary spins are confined within the $V_{G}$-defined quantum dots. **c** Conceptual illustration of the environment (gray dotted box) and the qubits. The Exchange-Time-Integral (ETI)—combining the exchange modulation (blue lines) and time (blue clock icon)—serves as the effective control parameter that determines the qubit evolution along a trajectory in Hilbert space. **d** Schematic representation of entanglement trajectories, where the horizontal gray line denotes the boundary separating the entangled (upper) and separable (lower) subspaces, highlighting distinct dynamical regimes including snake, bouncing, pulse, and boundary-residing trajectories.

As illustrated in Fig. 1a, the proposed device consists of two spin qubits, *A* and *B*, separated by a distance 2*R*, and a mediating environment. This environment comprises a central spin qudit *C* (a *d* -level quantum system with spin $\vec {S}^{C}$) and the itinerant electrons of a 2DEG, denoted as *e*. The central spin polarizes the 2DEG, generating a spin-density imbalance whose sign oscillates with distance—a hallmark of the RKKY interaction. We examine the entanglement between the two qubits mediated by this exchange. Coupling to the environment can be realized via two schemes: (i) motion-driven suspended qubits interacting with local electron spins in the 2DEG via proximity effects (Fig. 1a), or (ii) gate-driven stationary qubits confined by QDs in the 2DEG (Fig. 1b). As a possible proof-of-concept realization, the motion-driven scheme may be implemented using suspended qubits actuated by external electromagnetic fields, inducing lateral nanomechanical vibration that allows the qubits to sample different spatial values of the RKKY exchange. Alternatively, the gate-driven scheme may utilize high-mobility GaAs/AlGaAs heterostructures forming the 2DEG, with time-dependent gate voltages that define the QDs and modulate the RKKY exchange^44,72,73^. In both cases, the qubit states can be reconstructed using standard quantum-state tomography to experimentally extract the concurrence. Since both schemes generate an equivalent sign-alternating exchange, our subsequent analysis focuses on the case of suspended qubits.

The local *s*-*d* exchange interaction between the spins (qubits or qudits) and the conduction electrons is modeled using a Dirac delta function as

1 $$\begin{aligned} H_{sd}=J_{sd}\sum _{i\in \{A,B,C\}}\vec {S}^{i}\cdot \vec {\sigma }^{e}\,\delta ( \vec {r}^{e}-\vec {r}^{i})\text {,} \end{aligned}$$

which, upon integrating out the electron degrees of freedom, yields an effective indirect RKKY interaction among the localized spins:

2 $$\begin{aligned} H_{\textrm{RKKY}}=J(r^{AB})\,\vec {\sigma }^{A}\cdot \vec {\sigma } ^{B}+J(r^{AC})\,\vec {\sigma }^{A}\cdot \vec {S}^{C}+J(r^{BC})\,\vec {\sigma } ^{B}\cdot \vec {S}^{C}\text {,} \end{aligned}$$

where $\vec {r}^{ij}=\vec {r}^{i}-\vec {r}^{j}$. Here, $\vec {r}^{A}$, $\vec {r} ^{B}$, $\vec {r}^{C}$, and $\vec {r}^{_{e}}$ denote the position vectors of qubits *A* and *B*, qudit *C*, and the electron, respectively. The operators $\vec {\sigma }^{A}$ and $\vec {\sigma }^{B}$ represent the Pauli vector matrices of the qubit subsystems, while $\vec {S}^{C}$ is the spin operator of qudit *C* (where $\vec {S}^{C}=\vec {\sigma }^{C}/2$ for the $d=2$ case). Notably, the coupling strength scales as $J(r)\propto J_{sd}^{2}$ and, in $\alpha$ spatial dimensions, decays as $J(r)1\propto /r^{\alpha }$ (modulated by oscillations). Consequently, we neglect the direct coupling between *A* and *B*, assuming $J(r^{AB})\ll J(r^{AC})$ and $J(r^{AB})\ll J(r^{BC})$. The qubits are subject to a local, spin-independent orbital confinement potential $H_{o}$. The resulting spatial evolution $r^{AB}\left( t\right)$ governed by $H_{o}$ leads to an effective spin Hamiltonian of the form

3 $$\begin{aligned} H=J(r^{A}\left( t\right) )\,\vec {\sigma }^{A}\cdot \!\vec {S} ^{C}+J(r^{B}\left( t\right) )\,\vec {\sigma }^{B}\cdot \!\vec {S}^{C}\text {,} \end{aligned}$$

where we have set the origin at $\vec {r}^{C}\equiv 0$. The explicit time dependence of the RKKY exchange terms, $J(r^{A}\left( t\right) )$ and $J(r^{B}\left( t\right) )$, originates from the motion of the qubits. This dependence can be induced by a simple harmonic confinement potential,

4 $$\begin{aligned} H_{o}=\frac{1}{2}k^{A}\left( \vec {r}^{A}-\vec {R}_{0}^{A}\right) ^{2}+\frac{1 }{2}k^{B}\left( \vec {r}^{B}-\vec {R}_{0}^{B}\right) ^{2}\text {,} \end{aligned}$$

which drives the vibrational motion of *A* and *B* about their respective equilibrium positions, $\vec {R}_{0}^{\,A}$ and $\vec {R}_{0}^{\,B}$. However, any mechanism that induces a dynamic sign change in *J*^44,72,73^ will enable the design of desired entanglement trajectories, such as those shown in Fig. 1d. We note that an alternating exchange sign can equivalently be achieved by applying a harmonic potential to the environmental qudit *C*, rather than to *A* or *B*.

The operating regime considered here is governed by the competition between two mechanisms: the Kondo effect, which screens a local moment into a many-body singlet^76^, and the RKKY interaction, which produces an oscillatory ordering of local moments^41,45,49,77^. In this paper, we concentrate on the RKKY-dominated regime, characterized on the Doniach phase diagram by a sufficiently small exchange coupling $J_{sd}$—or, equivalently, a low density of states at the Fermi level, $N(E_{F})$. This condition ensures that the RKKY interaction scale, $J\sim J_{sd}^{2}N(E_{F})$ , exceeds the Kondo energy scale $k_{B}T_{K}$, where $T_{K}\sim \exp \!\left[ -1/\left( N(E_{F})J_{sd}\right) \right]$^78,79^.

We focus on the motion-driven scheme, where capital *R* denotes the locations of the exchange nodes (zeros of *J*). Specifically, we set the equilibrium positions $\vec {R}_{0}^{\,A}=\vec {R}_{n}$ and $\vec {R}_{0}^{\,B}= \vec {R}_{-n}$ such that the *A* and *B* qubits vibrate about the *n*-th and ( $-n$)-th nodes, respectively, satisfying $J(\vec {R}_{0}^{\,A/B})=0$. According to Eq. (4), the spatial evolution of the qubits, characterized by the vibrational motion parallel to the 2DEG plane, is given by

5 $$\begin{aligned} r^{A/B}\left( t\right) =R_{0}^{A/B}+\mathscr {R}^{A/B}\cos \left( \omega ^{A/B}t+\phi ^{A/B}\right) \text {,} \end{aligned}$$

with frequency $\omega ^{A/B}$, phase constant $\phi ^{A/B}$, and displacement amplitude $\mathscr {R}^{A/B}$. We assume small amplitudes $\mathscr {R}^{A/B}\ll \left| \vec {R}_{n+1}-\vec {R}_{n}\right|$ so that the exchange interaction in Eq. (3) can be linearized with respect to the distance $r^{A/B}$ as

6 $$\begin{aligned} J(r^{A/B}\left( t\right) )\approx J\left( R_{0}^{\,A/B}\right) +\left. \frac{ dJ\left( r^{A/B}\right) }{dr^{A/B}}\right| _{\vec {r}^{A/B}=\vec {R} _{0}^{A/B}}\times \left( r^{A/B}-R_{0}^{A/B}\right) \text {.} \end{aligned}$$

Substituting Eq. (5) into this expansion yields

7 $$\begin{aligned} J^{A/B}(t)=J_{0}^{A/B}\cos \left( \omega ^{A/B}t+\phi ^{A/B}\right) \text {,} \end{aligned}$$

with the alternating-exchange amplitude defined as

8 $$\begin{aligned} J_{0}^{A/B}=\left. \frac{dJ\left( r^{A/B}\right) }{dr^{A/B}}\right| _{r^{A/B}=\vec {R}_{0}^{A/B}}\mathscr {R}^{A/B}\text {.} \end{aligned}$$

Consequently, the *A*-*B* system in Eq. (3) effectively evolves under the time-dependent Hamiltonian,

9 $$\begin{aligned} H\left( t\right)= & J^{A}\left( t\right) \,\vec {\sigma }^{A}\cdot \!\vec {S} ^{C}+J^{B}\left( t\right) \,\vec {\sigma }^{B}\cdot \!\vec {S}^{C} \end{aligned}$$

10 $$\begin{aligned}= & J_{0}^{A}\cos \left( \omega ^{A}t+\phi ^{A}\right) \,\vec {\sigma } ^{A}\cdot \!\vec {S}^{C}+J_{0}^{B}\cos \left( \omega ^{B}t+\phi ^{B}\right) \,\vec {\sigma }^{B}\cdot \!\vec {S}^{C}\text {.} \end{aligned}$$

We elucidate the role of the dynamical exchange $J\left( t\right)$ when the two qubits vibrate at the same frequency $\omega ^{A}=\omega ^{B}=\omega$, specifically for the synchronous regimes of in-phase ($\Delta \phi \equiv \phi ^{A}-\phi ^{B}=0$) and antiphase ($\Delta \phi =\pm \pi$) motion. The dynamics of the total system comprising qubits *A*, *B*, and qudit *C* are described by the full (denoted by tilde) density matrix (DM) $\tilde{\rho } \left( t\right)$ governed by the Liouville-von Neumann equation,

11 $$\begin{aligned} \frac{d\tilde{\rho }\left( t\right) }{dt}=-i\left[ H\left( t\right) ,\tilde{ \rho }\left( t\right) \right] \text {,} \end{aligned}$$

where $\hbar \equiv 1$. The formal solution is

12 $$\begin{aligned} \tilde{\rho }\left( t\right) =U\left( t\right) \tilde{\rho }_{0}U^{\dagger }\left( t\right) \text {,} \end{aligned}$$

with the evolution operator

13 $$\begin{aligned} U\left( t\right) =\tau \left\{ \exp \left[ -i\int \nolimits _{0}^{t}H\left( t^{\prime }\right) dt^{\prime }\right] \right\} \text {,} \end{aligned}$$

and the initial DM $\tilde{\rho }\left( t=0\right) \equiv \tilde{\rho }_{0}$. Crucially, in these synchronous regimes, the time dependence in Eq. (10) becomes a common scalar factor, allowing the Hamiltonian to be factorized into a time-dependent amplitude and a time-independent operator. Consequently, the Hamiltonian commutes with itself at different times ($\left[ H\left( t\right) ,H\left( t^{\prime }\right) \right] =0$), allowing the time-ordering operator $\tau$ to be dropped.

Consider the in-phase case where $\phi _{A}=\phi _{B}\equiv \phi$. Direct integration yields

14 $$\begin{aligned} U\left( t\right) =\exp \left[ -iI\left( t\right) \times \sum _{k=x,y,z}\left( \sigma _{k}^{A}\eta _{J,k}^{A}+\sigma _{k}^{B}\eta _{J,k}^{B}\right) S_{k}^{C}\right] \end{aligned}$$

where the time-dependence is fully captured by the quantity

15 $$\begin{aligned} I\left( t\right) =\frac{J_{0}}{\omega }\left[ \sin \left( \omega t+\phi \right) -\sin \left( \phi \right) \right] \text {.} \end{aligned}$$

We refer to $I\left( t\right)$ as the ETI, which quantifies the accumulated exchange interaction^34^. Here, we have generalized to anisotropic exchange by defining the vector amplitudes

16 $$\begin{aligned} \vec {J}_{0}^{A/B}=\left( J_{0,x}^{A/B},J_{0,y}^{A/B},J_{0,z}^{A/B}\right) =J_{0}\vec {\eta }_{J}^{A/B}\text {.} \end{aligned}$$

We set the scaling such that $J_{0}\equiv \left| \vec {J} _{0}^{A}\right|$ and $\vec {\eta }_{J}^{A/B}$ is a unit vector. The coupling ratio is defined as $\gamma \equiv J_{0}^{A}/J_{0}^{B}$, implying the relative vector components are related by

17 $$\begin{aligned} \eta _{J,k}^{B}=\frac{\eta _{J,k}^{A}}{\gamma }\text {,} \end{aligned}$$

with $k\in \left\{ x,y,z\right\}$. For antiphase motion, the condition $\phi ^{A}=\phi ^{B}\pm \pi$ yields a negative coupling ratio ($\gamma <0$), as $\cos \left( \phi +\pi \right) =-\cos (\phi )$. Employing the eigen-decomposition of the time-independent operator,

18 $$\begin{aligned} \sum _{k=x,y,z}\left( \sigma _{k}^{A}\eta _{J,k}^{A}+\sigma _{k}^{B}\eta _{J,k}^{B}\right) S_{k}^{C}=VDV^{\dagger }\text {,} \end{aligned}$$

the DM evolution reduces to

19 $$\begin{aligned} \tilde{\rho }\left( t\right) =V\exp \left[ -iI\left( t\right) D\right] V^{\dagger }\times \tilde{\rho }_{0}^{{}}\times V\exp \left[ iI\left( t\right) D\right] V^{\dagger }\text {,} \end{aligned}$$

with *V* (*D*) the eigenvector (diagonal-eigenvalue) matrix. This result highlights that the exchange strength $J_{0}$ and time *t* influence the system solely through the single parameter $I\left( t\right)$. Time reversal is thus effected by reversing the sign of the exchange profile to retrace $I\left( t\right)$. The validity of this scalar ETI extends beyond the linearized harmonic motion shown above. Generally, as long as the ratio between the couplings remains constant in time—i.e., $\vec {J}^{A}\left( t\right) =\gamma \vec {J}^{B}\left( t\right)$—the Hamiltonian factorizes into a common time-dependent scalar factor and a static spin operator. In such cases, the ETI takes the general form, up to a constant,

20 $$\begin{aligned} I\left( t\right) =\int \limits _{0}^{t}J^{A/B}\left( t^{\prime }\right) dt^{\prime }\text {.} \end{aligned}$$

This factorization allows the complex dynamics to be mapped onto the single ETI, enabling the designable trajectories discussed below. Conversely, the out-of-phase case requires extending the ETI to a vector form, yielding irregular trajectories where reversing the exchange profile sign does not effect time reversal. This regime is detailed in Section "Out-of-phase vibrations". Physically, the calculations above assume the fast-motion limit, implying the qubit vibration frequency is sufficiently high that the spatial RKKY profile (determined by the electron spin polarization in the 2DEG) does not fundamentally reconfigure within one period. We further note that the constant exchange ratio $\gamma$, required for this Hamiltonian factorization, can be engineered via coherent control of external fields. This is achievable through a global time-varying gate voltage $V_{G}\left( t\right)$ in the gate-driven scheme, or via synchronized electromagnetic waves in the motion-driven scheme.

To quantify the deviation of a state from the *A*-*B* entanglement-separability boundary, we compute the reduced DM

21 $$\begin{aligned} \rho \left( t\right)\equiv &\, \rho ^{AB}\left( t\right) \nonumber \\= & \,Tr_{C}\left[ \tilde{\rho } \left( t\right) \right] \text {,} \end{aligned}$$

obtained by tracing out the spin degrees of freedom of qubit *C*. We then employ the concurrence $\mathscr {C}_{E}$^80–82^, *extended to admit negative values *,

22 $$\begin{aligned} \mathscr {C}_{E}(t)=2\kappa _{\max }-K \end{aligned}$$

where $\kappa \in \left\{ \kappa _{1},\kappa _{2},\kappa _{3},\kappa _{4}\right\}$ are the eigenvalues of the matrix

23 $$\begin{aligned} \sqrt{\sqrt{\rho \left( t\right) }\rho ^{\prime }\left( t\right) \sqrt{\rho \left( t\right) }}\text {,} \end{aligned}$$

with $$\kappa _{\max }$$$$=\max \left( \kappa \right)$$, and $$K=\left( \kappa _{1}+\kappa _{2}+\kappa _{3}+\kappa _{4}\right)$$. Here, $$\rho ^{\prime }\left( t\right)$$ is constructed from the complex conjugate $$\rho \left( t\right) ^{*}$$ via the transformation,

24 $$\begin{aligned} \rho ^{\prime }\left( t\right) =\sigma _{y}^{\otimes 2}\rho \left( t\right) ^{*}\sigma _{y}^{\otimes 2}\text {.} \end{aligned}$$

Importantly, positive values of $\mathscr {C}_{E}(t)$ indicate entanglement, while negative values indicate separability. A larger absolute value $\left| \mathscr {C}_{E}(t)\right|$ signifies a greater deviation from the boundary. As verified by our numerical calculations, this metric $\mathscr {C}_{E}(t)$ aligns qualitatively with the entanglement negativity^59^.

### Boundary-proximal initial states

Although Eq. 19 allows for an explicit expression of the evolved $\mathscr {C}_{E}(t)$ and predictions on the existence of ESD and ESB (see Supplementary Section S1) in the mixed-state case, to systematically explore the navigation of entanglement trajectories in both mixed and pure states, we employ the ISs characterized by the entanglement switch parameter (ESP^59^) $\varepsilon$. The ESP is defined as penetrable if it allows the IS to be tuned across the entanglement-separability boundary: $\varepsilon >0$ corresponds to entangled states, while $\varepsilon <0$ denotes separable states. By selecting ISs near $\left| \varepsilon \right| \approx 0$, we can investigate critical dynamics such as ESD and ESB, with the exact $\varepsilon =0$ corresponding to the states at the boundary. The initial reduced DM, $\rho _{0}\equiv \rho \left( t=0\right)$, is expanded in the basis of the four Bell states,

25 $$\begin{aligned} \left| \alpha ^{\pm }\right\rangle =\sqrt{\frac{1}{2}}\left| \uparrow ,\uparrow \right\rangle \pm \sqrt{\frac{1}{2}}\left| \downarrow ,\downarrow \right\rangle \end{aligned}$$

and

26 $$\begin{aligned} \left| \beta ^{\pm }\right\rangle =\sqrt{\frac{1}{2}}\left| \uparrow ,\downarrow \right\rangle \pm \sqrt{\frac{1}{2}}\left| \downarrow ,\uparrow \right\rangle \text {,} \end{aligned}$$

yielding the form

27 $$\begin{aligned} \rho _{0}=w_{\alpha ^{+}}\left| \alpha ^{+}\right\rangle \left\langle \alpha ^{+}\right| +w_{\alpha ^{-}}\left| \alpha ^{-}\right\rangle \left\langle \alpha ^{-}\right| +w_{\beta ^{+}}\left| \beta ^{+}\right\rangle \left\langle \beta ^{+}\right| +w_{\beta ^{-}}\left| \beta ^{-}\right\rangle \left\langle \beta ^{-}\right| \text {,} \end{aligned}$$

where the weighting $W=\left( w_{\alpha ^{+}},w_{\alpha ^{-}},w_{\beta ^{+}},w_{\beta ^{-}}\right)$ is normalized, $w_{\alpha ^{+}}+w_{\alpha ^{-}}+w_{\beta ^{+}}+w_{\beta ^{-}}=1$. The specific weightings considered are listed in the first column of Table 1, where nonzero weights are set near 1/2. For mixed states (Tr$[(\tilde{\rho }_{0})^{2}]<1$ ), we initialize the system as $\rho _{0}\otimes \left| \uparrow \right\rangle \left\langle \uparrow \right|$, with the central spin-1/2 *C* set to the spin-up state $\left| S^{C}\right\rangle =\left| \uparrow \right\rangle$. For pure states (Tr$[(\tilde{\rho }_{0})^{2}]=1$), we construct the IS as $\tilde{\rho }_{0}=\left| \psi _{0}\right\rangle \left\langle \psi _{0}\right|$, with

28 $$\begin{aligned} \left| \psi _{0}\right\rangle =\sum _{\begin{array}{c} i=\alpha ^{+},\alpha ^{-},\beta ^{+},\beta ^{-} \\ w_{i}\ne 0 \end{array}}\sqrt{w_{i}}\left| i^{C}\right\rangle \otimes \left| i\right\rangle \text {,} \end{aligned}$$

where the summation runs over the basis states $\left| i\right\rangle \in \left\{ \left| \alpha ^{\pm }\right\rangle ,\left| \beta ^{\pm }\right\rangle \right\}$ corresponding to the nonzero weights $w_{i}$ in Table 1. The environmental states $\left| i^{C}\right\rangle$ map to the spin-*z* eigenstates $\left| m\right\rangle$ of *C* arranged in descending order ($\left| m=S^{C}\right\rangle$, $\left| S^{C}-1\right\rangle$, $\cdots$, $\left| -S^{C}\right\rangle$). The spin $S^{C}$ is chosen to match the number of nonzero Bell components in the expansion: $S^{C}=1/2$ for $W_{1 \text {--}6}$, $S^{C}=1$ (qutrit) for $W_{7\text {--}10}$, and $S^{C}=3/2$ (qudit, $d=4$) for $W_{11\text {--}14}$. Generally, *ISs constructed* *from more than two Bell states* ($W_{7\text {--}14}$) *are* *penetrable*, i.e., facilitate boundary crossings (ESD/ESB) as $\varepsilon$ changes sign, whereas ISs limited to two Bell states ($W_{1 \text {--}6}$) typically exhibit ESD or TZD. For instance, all $W_{1\text {--} 6}$ *pure* states yield the TZD (see Table 1). As the trivial dynamics of the TZD do not result in boundary crossings, these cases are not examined in the following results.

## Results

In the simulation results presented below, bold italic letters *M* and *P* in the figures denote the cases of mixed and pure states, respectively; solid and dashed lines represent initially entangled states ($\varepsilon >0$) and initially separable states ($\varepsilon <0$), respectively. Unless otherwise specified, the following defaults are adopted in the numerical simulations. Isotropic exchange is assumed within the configuration shown in Fig. 1a [Eq. (5)], with identical vibrational frequencies ($\omega ^{A}=\omega ^{B}\equiv \omega$) and exchange strengths ($J_{0}^{A}=J_{0}^{B}\equiv J_{0}$), yielding $\gamma =1$. The qubit motion commences from a position outside the exchange nodes, with the phase at $\phi _{B}=0$. For in-phase and antiphase motions (Section "In-phase and antiphase vibrations"), the dynamics are governed by Eq. (19), while Eq. (13) describes the out-of-phase motion (Section "Out-of-phase vibrations"). All analytical results have been verified to match the direct numerical integration of the master equation [Eq. (11)]. The standard rectangle integration scheme with uniform time grid points is adopted, ensuring numerical convergence to an accuracy of approximately $10^{-4}$. Energies are in units of $\left| J_{0}\right| \equiv 1$, and time *t* in units of $\hbar /\left| J_{0}\right|$. To observe critical boundary dynamics (ESD, ESB, or TZD), the ESP $\left| \varepsilon \right| =0.01$ is selected, with $J_{0}=-1$. The characteristic operating period $T^{*}$ is determined from the time $t^{*}$ required for the state to reach the entanglement-separability boundary

29 $$\begin{aligned} \mathscr {C}_{E}(t^{*})=0, \end{aligned}$$

where the relation is given by

30 $$\begin{aligned} t^{*}=T^{*}/4\text {.} \end{aligned}$$

The resulting values of $T^{*}$ (and the corresponding frequency $f=1/T^{*}$) for the selected $\varepsilon$ are listed in Table 1.

Note that the RKKY-driven spin dynamics can be disrupted by ferromagnetic resonance (FMR) in the few-GHz range, where resonant energy absorption enhances spin precession. To ensure that the RKKY exchange remains the dominant mechanism, operation away from this resonance regime is required. This can be achieved by reducing the vibrational amplitude or using a proximate 2D Fermi-sea substrate (Fig. 1b) yielding $\left| J_{0}\right| \lesssim 1$ $\mu$eV; this upper limit corresponds to a characteristic frequency $\left| J_{0}\right| /\hbar \approx 1.52\times 10^{9}$ rad/s (or equivalently, 0.24 GHz), which is well separated from the FMR band. Furthermore, bearing in mind that sign changes in $J^{A/B}\left( t\right)$ signify time and trajectory reversal, we can tailor the entanglement profile as demonstrated below.

### In-phase and antiphase vibrations

This section examines the entanglement trajectories for in-phase and antiphase vibrations. Because the underlying analysis is identical in both cases, as the exchange-time integral (ETI) applies equally, we restrict the discussion to the in-phase case. For mixed states, the characteristic period $T^{*}$, defined by $\mathscr {C}_{E}(T^{*}/4)=0$, can be estimated analytically in the short-time regime as detailed in Supplementary Section S2. Substituting $T^{*}$ into Eq. (15) yields the weight-dependent characteristic ETI,

31 $$\begin{aligned} I_{W}^{*}\equiv I\left( T^{*}/4\right) \text {.} \end{aligned}$$

**Figure 2:**
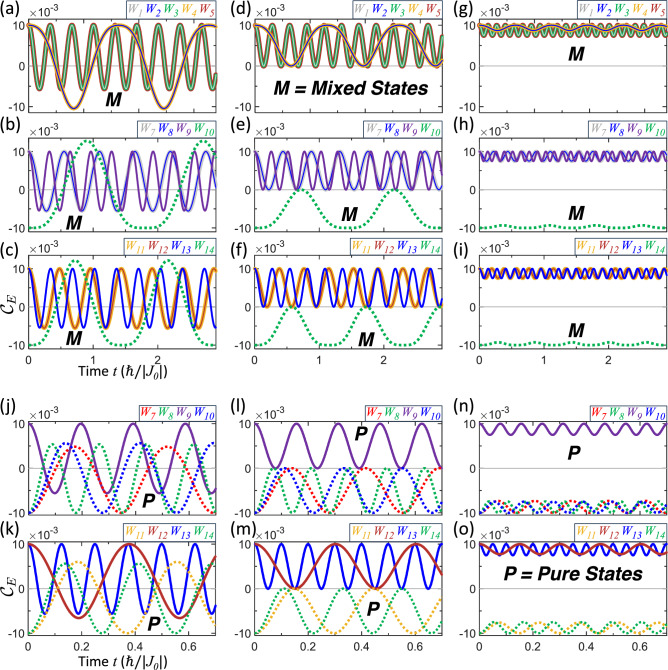
Entanglement trajectories for mixed (marked by bold italic *M*) and pure (marked by bold italic *P*) states under in-phase vibrations. The extended concurrence $\mathscr {C}_{E}(t)$ is plotted versus time *t* for various Bell-state weightings $W_{1\text {--}14}$ (see corresponding color text labels). The upper panels **a**–**i** display the dynamics for mixed states: **a**–**c** show snake trajectories ($T=1.25$ $T^{*}>T^{*}$), **d**–**f** show bouncing trajectories ($T=T^{*}$), and **g**–**i** exhibit entangled/separable trajectories ($T=0.5$ $T^{*}<T^{*}$). The lower panels **j**–**o** show the corresponding dynamics for pure states: snake trajectories in **j** and **k**, bouncing trajectories in **l** and **m**, and entangled/separable trajectories in **n** and **o**. The solid lines denote initially entangled states ($\varepsilon >0$), while dashed lines denote initially separable states ($\varepsilon <0$).

Since the entanglement dynamics depend solely on the ETI, any exchange dynamics—whether or not it involves vibrations—described by the general expression (20) in Eq. (19), with the same initial weight *W* and an ETI satisfying $I_{W}^{*}$, will necessarily reach the entanglement–separability boundary at $t=T^{*}/4$. The trajectories can therefore be systematically engineered through frequency control. For vibrations with $T=T^{*}$, the ETI indicates a trajectory reversal at $t=T^{*}/4$. These trajectories correspond to reversible navigation, in which the qubit-state evolution is governed by the periodic accumulation and unwinding of the ETI, so that reversing the sign of $J\left( t\right)$ effectively retraces the entanglement history. Consequently, oscillatory trajectories arise for $T\gtrsim T^{*}$, $T=T^{*}$, and $T\lesssim T^{*}$, corresponding to snake, bouncing, and entangled- or separable-confined profiles, respectively. The snake trajectory (for slightly larger $T\gtrsim T^{*}$) periodically crosses the entanglement-separability boundary. The bouncing trajectory (for $T=T^{*}$) reverses immediately upon reaching the boundary, corresponding to a TZD. The entangled/separable trajectory (for slightly smaller $T\lesssim T^{*}$) remains confined to one subspace without crossing the boundary. These trajectories are shown in Fig. 2 for both mixed and pure states.

For entanglement trajectories based on non-periodic motion, we consider the same vibrational scenario, except that the qubit motion is abruptly halted upon reaching the exchange nodes. The exchange coupling follows Eqs. (5) and (7), now modulated by a unit-step function $\Theta$,

32 $$\begin{aligned} J^{A/B}(t)=J_{0}\cos \left( \frac{2\pi t}{T^{*}}\right) \Theta \left( \frac{T^{*}}{4}-t\right) \text {.} \end{aligned}$$

**Figure 3:**
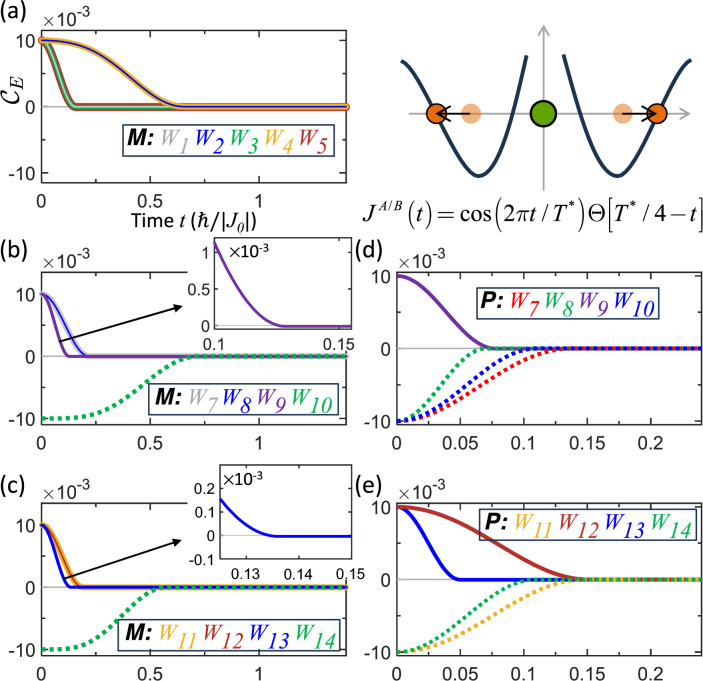
Entanglement trajectories originating from abruptly halted vibrational motion (schematic in the upper right) for (**a**–**c**) mixed states and (**d**–**e**) pure states with different weightings. The qubits remain at the entanglement-separability boundary after $t\ge T^{*}/4$, producing a boundary-residing trajectory. The insets show zoomed views near the stopping time $t=T^{*}/4$. All trajectories approach the boundary tangentially (smoothly), despite the abrupt cessation of motion.

Figure 3 shows the resulting trajectories for both mixed and pure states. After *t* $\ge$ $T^{*}/4$, the qubits remain pinned at the entanglement-separability boundary, forming a boundary-residing trajectory. Notably, although the motion stops abruptly, the entanglement exhibits a smooth approach to zero $\left. d\mathscr {C} _{E}(t)/dt\right| _{t=T^{*}/4}=0$ in Fig. 3. As a result, neither ESD nor ESB occurs. This demonstrates that an abrupt halt of the qubit motion, even involving a kinetic discontinuity (abrupt change in velocity), does not necessarily induce sudden entanglement dynamics.

**Figure 4:**
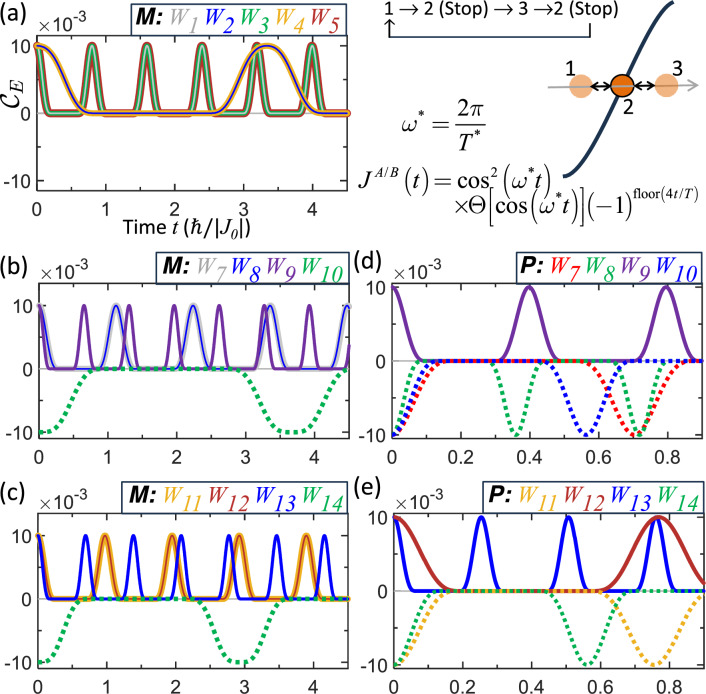
Entanglement pulses generated by controlled stopping and restarting of qubit cyclic motion, as shown schematically in the upper-right panel. The qubits halt temporarily at the exchange nodes, producing entangled (solid) and separable (dashed) pulses. Panels **a**–**c** show mixed states, while panels **d** and **e** show pure states. The trajectories include repeated boundary-residing segments, with the qubits departing from and returning to the boundary between these segments, thereby forming pulse trains.

Alternatively, the boundary-residing behavior is also accessible by employing a smooth (continuous-velocity) stop, where the exchange in Eq. (32) follows $\cos ^{2}(\theta )$ instead of cos$\left( \theta \right)$. In this case, $T^{*}$ is recomputed according to Eqs. (29) and (30). Specifically, this protocol realizes a pulse trajectory by employing the time dependence

33 $$\begin{aligned} J^{A/B}(t)=J_{0}\cos ^{2}\left( \frac{2\pi t}{T^{*}}\right) \Theta \left( \cos \left( \frac{2\pi t}{T^{*}}\right) \right) \times \left( -1\right) ^{floor\left( 4t/T^{*}\right) }\text {,} \end{aligned}$$

which corresponds to a temporary dwell of the motion whenever the qubit reaches an exchange node (see the schematics in Fig. 4 ). Here, the function *floor* denotes rounding toward negative infinity. Figure 4 shows the resulting pulse trajectories for both mixed and pure states obtained from Eq. (33). The dwell duration is $T^{*}/2$, spanning the intervals $\left( 2p+1\right) T^{*}/4$ to $\left( 2p+2\right) T^{*}/4$ with $p=0,1,2,\ldots$. This sequence produces repeated boundary-residing segments, generating entangled (solid lines) and separable (dashed lines) pulse trains. A single pulse can be produced by imposing a permanent stop.

We note, however, that for boundary-residing and pulsed trajectories, quantum position fluctuations $\delta r$ prevent the qubit from being fixed exactly at the exchange node. Such fluctuations can be mitigated using position-squeezed states^83–86^, ensuring that $\delta r$ remains much smaller than the vibration amplitude. A finite positional uncertainty $\delta r$ around the exchange node induces a residual exchange fluctuation $\delta J\approx \left( dJ/dr\right) \delta r$. Within the linearized model, $\delta J/J_{0}\approx \delta r/\mathscr {R}$; thus, for $\delta r\approx 1\,\textrm{nm}$ and $\mathscr {R}\approx 100\,\textrm{nm}$ (a typical gate-tunable Fermi wavelength in a 2DEG), the induced exchange deviation is about 1%. Because the short-time growth of the extended concurrence scales as $J^{2}$^59^, this corresponds to a residual $\delta \mathscr {C}_{e}$ of order 0.01%. In practice, for entangled (separable) pulses, one may choose a slightly larger $T^{*}$ so that the fluctuation in the extended concurrence $\delta \mathscr {C}_{e}$ remains safely below (above) the boundary. Therefore, in the motion-driven scheme, the boundary-residing trajectory should be understood as an approximate trajectory with small residual fluctuations around the boundary, as required by the uncertainty principle.

**Figure 5:**
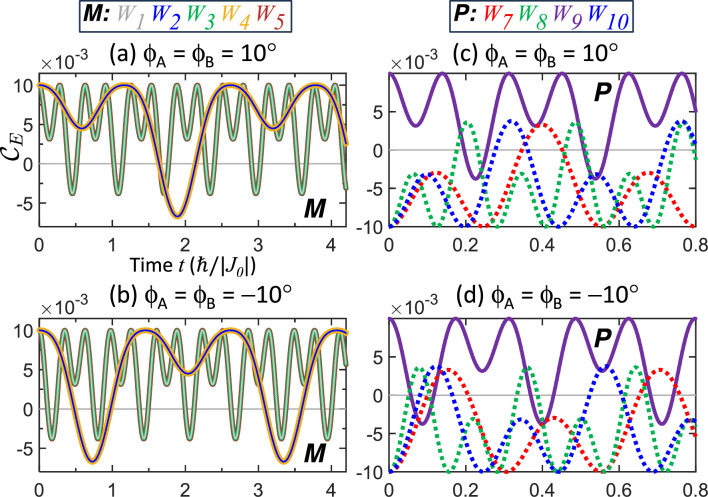
Entanglement trajectories for qubits initialized with a finite vibrational phase $\phi \ne 0$. Qubits vibrating toward ($\phi =10{{}^\circ }$) or away ($\phi =-10{{}^\circ }$) from the exchange nodes with period $T=T^{*}$ in Table 1 produce snake trajectories. Panels **a** and **b** show mixed states with weightings $W_{1\text {--}5}$, and panels **c** and **d** show pure states with weightings $W_{7\text {--}10}$. A finite $\phi$ causes asymmetric vertical shifts, while reversing its sign introduces horizontal phase shifts. Solid and dashed lines represent positive ($\varepsilon >0$) and negative ($\varepsilon <0$) ESP, respectively.

When qubits are initialized closer to the exchange nodes, a finite vibrational phase $\phi \ne 0$ can be introduced. This modification preserves the tunability of the trajectories via the period but introduces asymmetry in the entanglement evolution. As shown in Fig. 5, using $T=T^{*}$ from Table 1, similar snake trajectories emerge for selected weightings: $W_{1\text {--}5}$ for mixed states and $W_{7\text {--}10}$ for pure states. To understand the origin of the snake profile, we compare it to the standard ($\phi =0$) bouncing trajectories. While the vibrational amplitude is identical, the finite phase implies that the qubits are initialized closer to the exchange node. Consider an entangled IS with $\phi >0$. After $t=0$, the interval before the qubits reach the node is shortened, leading to a reduced accumulation of the ETI and a correspondingly weaker loss of entanglement. This initial phase therefore produces a shallow reversal entirely within the entangled subspace. Subsequently, after passing through the node, the qubits traverse the full range of motion until returning to the node. The ETI accumulated during this interval matches the standard bouncing case and exceeds the initial minor reduction. Consequently, this second swing drives the trajectory across the boundary into the separable subspace, giving rise to the characteristic snake profile. By the same reasoning, this snake trajectory is also realized for separable ISs.

In other words, a finite $\phi$ induces asymmetric vertical shifts, reflecting the difference in the ETI accumulation between consecutive entanglement recoveries. By contrast, reversing the sign of $\phi$ means the qubits initially move in the opposite direction; this effectively introduces a horizontal phase shift, as observed by comparing panels (a) with (b) and (c) with (d) in Fig. 5. The same argument applies to all other weightings exhibiting ESD and ESB listed in Table 1. For brevity, only the representative cases (mixed states with $W_{1\text {--}5}$ and pure states with $W_{7\text {--}10}$) are shown in the subsequent figures.

Importantly, for both in-phase and antiphase motion, we emphasize that the proposed device—characterized by alternating exchange signs—possesses built-in error correction against dephasing for oscillatory trajectories, Figs. 2, 4, and 5. Phase coherence is preserved because the rapid phase accumulation during the ferromagnetic ($J<0$) intervals is compensated by the rapid phase unwinding during the antiferromagnetic ($J>0$) intervals, refocusing the state and producing an echo-like correction^32,75^. Notably, being exchange-free, the node serves as an ideal location for qubits to idle. Conversely, prior to applying the confinement potential that yields the vibrations, the stable ground state resides at either the local maximum or minimum of *J*, making these points natural sites for entanglement initialization and development. While in-phase motion provides a robust baseline for tailoring entanglement dynamics, the strict periodicity of the evolution limits the accessible state space, particularly at short operation times. To overcome this limitation and access larger entanglement values, we now turn to the out-of-phase configuration, where the vibrational phases are shifted relative to one another.

### Out-of-phase vibrations

The introduction of a relative vibrational phase difference $\Delta \phi =\phi ^{A}-\phi ^{B}\ne 0$ defines the regime of out-of-phase vibrations. In this regime, the time-evolution operator can no longer be parameterized by a single ETI $I\left( t\right)$, as was done in Eq. (14). Instead, the dynamics are governed by two distinct integrals, $I^{A}\left( t\right)$ and $I^{B}\left( t\right)$. Although these integrals share the same functional form, their relative phase mismatch prevents the DM in Eq. (19) from retaining the strict periodic structure associated with a unified $I\left( t\right)$. Consequently, the resulting entanglement evolution becomes generally non-periodic.

**Figure 6:**
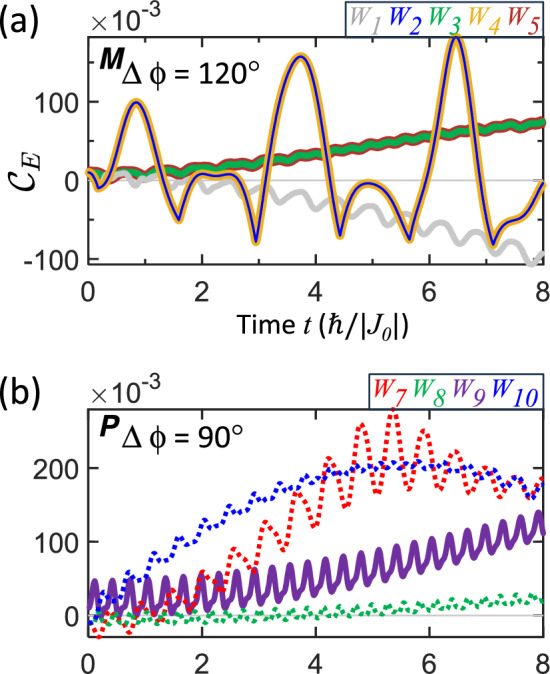
Out-of-phase entanglement trajectories for **a** mixed states with $\Delta \phi =120{{}^\circ }$ and **b** pure states with $\Delta \phi =90{{}^\circ }$. Solid lines represent initially entangled states with positive ESP, while dashed lines represent initially separable states with negative ESP. Vibration periods $T=T^{*}$ from Table 1 are used. The curves illustrate how out-of-phase motion drives the entanglement trajectories away from the boundary (horizontal gray lines).

Indeed, as shown in Fig. 6, the concurrence ceases to follow the simple repeating patterns observed in the in-phase or antiphase cases. Panels (a) and (b) illustrate the evolution for mixed and pure states, with phase differences of $\Delta \phi =120 {{}^\circ }$ and $\Delta \phi =90 {{}^\circ }$, respectively. The trajectories tend to drift progressively farther from the entanglement-separability boundary, exhibiting a slow overall growth upon which fast modulations (sub-oscillations) are superimposed. Notably, weightings $W_{3}$ and $W_{5}$ (mixed states) and $W_{7\text {--}10}$ (pure states) achieve significantly higher entanglement values than their $\Delta \phi =0$ counterparts. Furthermore, $W_{2}$ and $W_{4}$ (mixed states) exhibit swings with growing amplitude that cross the boundary multiple times. This complex behavior arises from the interference between the distinct accumulation rates of $I^{A}\left( t\right)$ and $I^{B}\left( t\right)$.

Figure 6 demonstrates that out-of-phase motion enables access to quantum states and high-entanglement subspaces that are inaccessible under strictly periodic motion ($\phi ^{A}=\phi ^{B}$). This regime highlights the potential of phase-engineered, alternating RKKY coupling to create highly versatile and non-repetitive entanglement trajectories. However, a key limitation in this scenario is that the trajectories do not naturally converge to a stable value, restricting their direct applicability for state preparation. To address this, we introduce damping mechanisms that provide a route to stabilize the trajectories.

In the out-of-phase regime where $\phi ^{A}\ne \phi ^{B}$, the exchange integrals $I^{A}\left( t\right)$ and $I^{B}\left( t\right)$ are generally not proportional. However, the introduction of damping fundamentally alters this behavior. Unlike the previous cases, damping causes the vibrational amplitude to decay, driving each ETI to converge to a distinct fixed value, $I^{A/B}\left( t\rightarrow \infty \right)$, as the qubits eventually settle at the exchange node ($J=0$). As a result, the system enters a frozen regime, stabilizing the generated entanglement. For the case of isotropic exchange modeled by $J^{A/B}(t)=J_{0}^{{}}\cos \left( \omega ^{A/B}t+\phi ^{A/B}\right) \exp \left( -\eta *t\right)$, the ETI can be expressed using Eq. (20) as

34 $$\begin{aligned} I^{A/B}\left( t\right) =\frac{-J_{0}}{\eta ^{2}+\omega ^{2}}F^{A/B}\left( \omega ,\eta ,t\right) \exp \left( -\eta *t\right) +\frac{J_{0}}{\eta ^{2}+\omega ^{2}}F^{A/B}\left( \omega ,\eta ,0\right) \text {,} \end{aligned}$$

where the function $F^{A/B}\left( \omega ,\eta ,t\right)$ is defined as

35 $$\begin{aligned} F^{A/B}\left( \omega ,\eta ,t\right)= & \,\eta \cos (\omega t+\phi ^{A/B})-\omega \sin \left( \omega t+\phi ^{A/B}\right) \nonumber \\= & \, \sqrt{\eta ^{2}+\omega ^{2}}\times \cos \left[ \omega t+\phi ^{A/B}+\arctan \left( \frac{\omega }{\eta }\right) \right] \text {.} \end{aligned}$$

Here $\eta$ represents the damping strength in units of $\left| J_{0}\right| /\hbar$. To streamline the analysis, we introduce the amplitude scaling factor,

36 $$\begin{aligned} \Gamma \equiv \frac{J_{0}}{\sqrt{\eta ^{2}+\omega ^{2}}}\text {,} \end{aligned}$$

and the shifted phase,

37 $$\begin{aligned} \Phi ^{A/B}\equiv \phi ^{A/B}+\arctan \left( \frac{\omega }{\eta }\right) \text {.} \end{aligned}$$

With these definitions, Eq. (34) simplifies to

38 $$\begin{aligned} I^{A/B}\left( t\right) =-\Gamma \cos \left( \omega t+\Phi ^{A/B}\right) \exp \left( -\eta *t\right) +I^{A/B}\left( t\rightarrow \infty \right) \text {,} \end{aligned}$$

where the ETI asymptotically approaches the constant stationary value,

39 $$\begin{aligned} I^{A/B}\left( \infty \right) =\Gamma \cos \left( \Phi ^{A/B}\right) \text {.} \end{aligned}$$

Since the cosine function is bounded by unity (i.e., $-1\le \cos \left( \omega t+\Phi \right) \le 1$), the ETI satisfies the inequality,

40 $$\begin{aligned} & \Gamma \cos \left( \Phi ^{A/B}\right) -\left| \Gamma \right| \exp \left( -\eta *t\right) \nonumber \\ & \le I^{A/B}\left( t\right) \nonumber \\ & \le \Gamma \cos \left( \Phi ^{A/B}\right) +\left| \Gamma \right| \exp \left( -\eta *t\right) \text {.} \end{aligned}$$

This bound offers valuable physical insights. First, it shows that weaker damping (smaller $\eta$) and lower vibration frequencies (smaller $\omega$ ) expand the accessible range of the ETI by increasing the magnitude of $\Gamma$. Accordingly, this expansion allows for greater achievable entanglement. Second, by comparing Eq. (40) with Eq. (39), we observe that $I^{A/B}\left( t\right)$ can transiently exceed its stationary value $I^{A/B}\left( \infty \right)$, particularly when $\eta$ is small. Accordingly, the transient entanglement $\mathscr {C} _{E}(t)$ can surpass the final stabilized value $\mathscr {C}_{E}(\infty )$. Thus, by tuning $\omega$ and $\eta$, one can engineer the dynamics to target a specific, stable value of entanglement.

**Figure 7:**
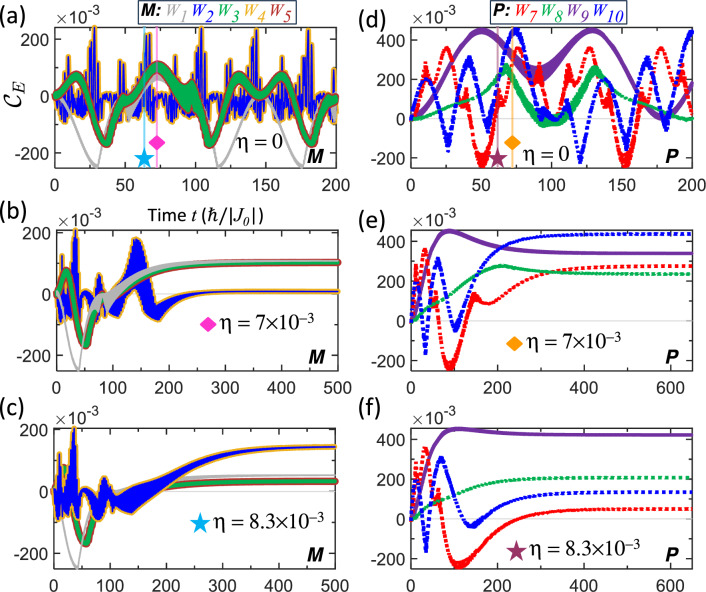
Entanglement evolution for **a**–**c** mixed states and **d**–**f** pure states with out-of-phase vibrations at $\Delta \phi =30 {{}^\circ }$. The vibration period $T=T^{*}$ from Table 1 is used. The undamped cases ($\eta =0$) are shown in **a** and **d**. The damped cases are shown with damping strength $\eta =7\times 10^{-3}$ (in units of $\left| J_{0}\right| /\hbar$) in (**b**) and (**e**) (diamond markers), and $\eta =8.3\times 10^{-3}$ in (**c**) and (**f**) (star markers). The vertical lines in **a** and **d** indicate the corresponding stabilization time $t_{s}$ observed in the damped regimes. The transient band-like curves arise from fast and closely spaced entanglement modulations. A weaker damping strength leads to a larger postponement of $t_{s}$. Under damping, time effectively slows down, causing the trajectories to appear stretched before $t_{s}$ and ultimately resulting in a time-frozen trajectory. For example, $W_{7}$ in **d** shows a minimum around $t=50$, while in **e** and **f** this minimum occurs later, around $t=100$.

The features of this damped evolution are illustrated in Fig. 7 for a representative phase difference of $\Delta \phi =30 {{}^\circ }$, where panels (a)–(c) and (d)–(f) display results for mixed and pure states, respectively. Panels (a) and (d) present the undamped reference cases, while the subsequent panels introduce damping with $\eta =7\times 10^{-3}$ (diamond markers) and slightly stronger damping with $\eta =8.3\times 10^{-3}$ (star markers). These values are chosen as tuning parameters to ensure that the stabilization takes place in a desired high-entanglement region of the trajectory. Clearly, decreasing the damping strength delays the stabilization time $t_{s}$—the characteristic time scale beyond which the entanglement becomes effectively fixed. This delay is evident when comparing the damped panels (b) with (c) and (e) with (f), with the vertical lines in panels (a) and (d) marking the corresponding onset $t_{s}$ of the frozen regime observed in the damped cases. In the early dynamics ($t<t_{s}$), the rapid entanglement modulations are so dense that they appear as continuous bands. During this phase, the trajectory profile retains the overall shape of the undamped case but with reduced amplitude and a slower effective evolution. However, the damping also causes the evolution to slow down, effectively stretching the profile along the time axis. For example, the first local minimum of $W_{3}$ in the undamped motion (a) occurs around $t=30$, whereas in the damped motion (b) and (c), the corresponding first local minimum appears later, after $t=45$. This delay reflects the slower accumulation of both $I^{A}\left( t\right)$ and $I^{B}\left( t\right)$, and thus the evolution, which eventually culminates in a time-frozen trajectory.

Accordingly, by optimizing the damping strength, one can maximize the final stable entanglement. This optimization relies on selecting an $\eta$ that targets a specific $t_{s}$ where the trajectory reaches a desired large entanglement. As shown in Fig. 7, specific weightings—$W_{1}$, $W_{3}$, $W_{5}$ in (b); $W_{2}$, $W_{4}$ in (c); $W_{7}$, $W_{8}$, $W_{10}$ in (e); and $W_{9}$ in (f)—yield frozen entanglement values that significantly exceed those achievable in the $\Delta \phi =0$ case. We note that $I^{A/B}\left( t\right)$ in Eq. (38) reduces to Eq. (15) only in the limit $\eta =0$ and $\Delta \phi =0$. In the damped, out-of-phase scenario, $I^{A}\left( t\right)$ is no longer proportional to $I^{B}\left( t\right)$, marking a fundamental departure from the single-ETI eigen-decomposition used in Eq. (19). Physically, this damping model is also realizable in a scenario where the two qubits, *A* and *B*, move in opposite directions away from the central qudit *C*, thereby experiencing a spatially decaying, alternating RKKY interaction.

Finally, we comment on the robustness of these trajectories against fluctuations in the coupling ratio $\gamma$. In the synchronous regime, the scalar ETI framework remains a good approximation under weak perturbations; according to the Baker-Campbell-Hausdorff expansion, the leading-order deviations arising from the non-commutativity of the qubit couplings scale as $J_{0}^{2}t^{2}$. Such errors are naturally suppressed in the fast-motion limit. Moreover, the scalar ETI description remains valid under common-mode perturbations, such as global gate-voltage fluctuations, because they preserve the proportionality between the couplings and hence the factorized structure of the Hamiltonian. In the damped out-of-phase regime, although noise may shift the precise damping strength $\eta$ that maximizes the entanglement, a practical mitigation is to choose a slightly larger $\eta$. This allows the trajectory to be frozen into a stationary state before accumulated errors become appreciable, at the cost of not necessarily reaching the maximum entanglement.

**Summary of Results.** Overall, the results show that the ETI enables systematic navigation of diverse entanglement trajectories, including snake, bouncing, boundary-residing, and pulsed profiles. In addition, out-of-phase motion combined with damping provides a practical route to freeze and stabilize large entanglement, thus complementing the reversible ETI-based control available in the synchronous regime.

## Conclusions

In conclusion, to navigate qubit entanglement trajectories, we propose an RKKY-based platform, Fig. 1, in which two spin qubits, *A* and *B*, couple to a central spin qudit *C* that induces an oscillatory spin polarization in the surrounding conduction electrons. To quantify the deviation from the entanglement-separability boundary, the concurrence is extended to include negative values; this extension enables a unified description of boundary-crossing dynamics. For qubits experiencing alternating exchange *J* around the node, the linearization (6) captures the relevant dynamics, yielding an effective time-dependent Hamiltonian (9). The qubit spatial motion is mapped onto the ETI through the time dependence of the exchange interaction. The ETI (20), obtained by integrating the time-dependent exchange, acts as a single experimentally accessible control variable that parameterizes the state evolution via (19). With our interest placed on the spin-qubit entanglement rather than on the environmental qudit and conduction electrons, the orbital or spatial degrees of freedom are traced out, preserving the form of the ETI-ruled evolution for any spin-independent $H_{o}$.

We identify two complementary dynamical regimes, cyclic and non-cyclic navigation. First, cyclic navigation corresponds to periodic motion, enabling the recurrent restoration of the state. Specifically, for in-phase and anti-phase vibrations under a harmonic confinement potential, the ETI reduces to (15). Using weighted Bell-state DMs, Eq. (27), we demonstrate frequency-controlled transitions between snake, bouncing, and boundary-residing trajectories for both mixed states and pure states (Figs. 2 and 3). The FMR can be avoided by selecting realistic exchange values that yield the operating frequencies listed in Table 1; the corresponding ETI values are computed (see Supplementary Section S2). By allowing temporary dwells at the nodes, entanglement pulse trains can be generated, with pulse separation controlled by the dwell duration, as shown in Fig. 4. A nonzero equal vibrational phase, $\phi =\phi ^{A}=\phi ^{B}$, induces an asymmetric vertical shift, while reversing its sign, $\phi \rightarrow -\phi$, produces a horizontal displacement of the trajectory (Fig. 5).

Second, non-cyclic navigation arises from out-of-phase exchange modulation. In this regime, the entanglement trajectory becomes non-periodic and is driven away from the boundary (Fig. 6), enabling access to higher entanglement values but without essential stabilization. We employ damping to stabilize the dynamics: the exchange-time integral gradually converges, slowing the evolution and ultimately freezing the system at a fixed entanglement value, as illustrated in Fig. 7. This final fixed entanglement is tunable through the damping strength, phase difference, and vibrational frequencies, as indicated by the converged ETI given in Eq. (39). Together, cyclic and damped non-cyclic regimes provide complementary control strategies for reversible manipulation and robust state preparation, respectively.

In addition, the advantages of the proposed device are as follows. The system is scalable for pairwise entanglement between qubits $A_{q}$ and $A_{q+1}$. For scalability, one relabels $A\rightarrow A_{1}$, $B\rightarrow A_{2}$, and $C\rightarrow C_{1}$ and then repeats the structure to form a chain $A_{1}$-$C_{1}$-$A_{2}$-$C_{2}$-$A_{3}\cdots$-$A_{Q-1}$-$C_{Q-1}$-$A_{Q}$ consisting of *Q* qubits. The eigenvalue decomposition (18) then becomes

$$\begin{aligned} \sum \limits _{q=1}^{Q}\sum _{k=x,y,z}\left( \sigma _{k}^{A_{q}}\eta _{J,k}^{A_{q}}\right) S_{k}^{C_{q}}=VDV^{\dagger }\text {,} \end{aligned}$$

with $\eta _{J,k}^{A_{1}}=\gamma _{2}\eta _{J,k}^{A_{2}}=\cdots =\gamma _{Q}\eta _{J,k}^{A_{Q}}$, and the same line of argument based on the ETI ( 20) remains applicable provided the configuration is symmetric, i.e., all neighboring qubit pairs experience the same time profile of *J*. In the present chain discussion, second-neighbor RKKY contributions are assumed negligible. For more general multi-mediator chains with appreciable superposed RKKY contributions, the persistence of a single scalar ETI is not guaranteed and is left for future work.

Compared with cavity-mediated entanglement schemes, the present approach is implemented in a direct on-chip solid-state platform and uses the ETI to provide transparent control over the entanglement evolution. Moreover, partial correction of errors due to dephasing is integrated into the oscillatory trajectories. In particular, the sign-alternating RKKY exchange itself naturally yields forward ($J>0$) and backward ($J<0$) evolution regions, in contrast to dynamical-decoupling-based schemes that rely on externally applied high-precision pulse sequences. The nodes ($J=0$) offer ideal locations for qubit information storage, while the shapeable trajectories enable the design of quantum encryption protocols and gate operations based on finite or repeated entanglement survival within specific time windows, achieving on-demand entanglement. In quantum sensing and metrology, the boundary-residing trajectory can be used for highly precise measurements of external observables that perturb the qubit toward or away from the entangled regime. The out-of-phase damping permits the establishment and maintenance of strong entanglement. Accordingly, the presented approach supports practical quantum devices that are both efficient, by allowing efficient entanglement generation, and stable, through the use of the exchange node and damping-assisted stabilization, thereby advancing computation, secure communication, and sensing with entanglement as a tunable resource.

**Table 1:** Bell-state weightings ($W_{1}\text {--}W_{14}$) and characteristic times $T^{*}$ ($\hbar /J_{0}$) for mixed and pure states with $\Delta \phi =0$. Depending on the initial weighting, the dynamics near $t\approx 0$ exhibit entanglement sudden death (ESD), sudden birth (ESB), or transition of zero duration (TZD). The *penetrable* entanglement switch parameter $\varepsilon$ sets whether the system begins in the entangled regime ($\varepsilon >0$, as in ESD), separable regime ($\varepsilon <0$, as in ESB), or on the boundary ($\varepsilon =0$).

$$\begin{array}{l} \mathrm{Weighting} \\ \left(w_{\alpha^{+}}, w_{\alpha^{-}}, w_{\beta^{+}}, w_{\beta^{-}}\right) \end{array}$$	$$\begin{array}{l} T^{*} (\hbar /J_{0})\ \mathrm{for} \\ \text{Mixed States} \end{array}$$	$$\begin{array}{l} \text{Dynamics of} \\ \text{Mixed States} \\ \mathrm{Near}\ t \approx 0 \end{array}$$	$$\begin{array}{l} T^{*} (\hbar /J_{0})\ \mathrm{for} \\ \text{Pure States} \end{array}$$	$$\begin{array}{l} \text{Dynamics of} \\ \text{Pure States} \\ \mathrm{Near}\ t\approx 0 \end{array}$$
$W_{1}=\left( \frac{1+\varepsilon }{2},\frac{1-\varepsilon }{2},0,0\right)$	0.6285	ESD	N/A	TZD
$W_{2}=\left( \frac{1+\varepsilon }{2},0,\frac{1-\varepsilon }{2},0\right)$	2.6185	ESD	N/A	TZD
$W_{3}=\left( \frac{1+\varepsilon }{2},0,0,\frac{1-\varepsilon }{2}\right)$	0.6283	ESD	N/A	TZD
$W_{4}=\left( 0,\frac{1+\varepsilon }{2},\frac{1-\varepsilon }{2},0\right)$	2.6185	ESD	N/A	TZD
$W_{5}=\left( 0,\frac{1+\varepsilon }{2},0,\frac{1-\varepsilon }{2}\right)$	0.6283	ESD	N/A	TZD
$W_{6}=\left( 0,0,\frac{1+\varepsilon }{2},\frac{1-\varepsilon }{2}\right)$	N/A	TZD	N/A	TZD
$W_{7}=\left( \frac{1+\varepsilon }{2},\frac{1-\varepsilon }{4},\frac{ 1-\varepsilon }{4},0\right)$	0.8801	ESD	0.5525	ESB
$W_{8}=\left( 0,\frac{1+\varepsilon }{2},\frac{1-\varepsilon }{4},\frac{ 1-\varepsilon }{4}\right)$	0.8779	ESD	0.2822	ESB
$W_{9}=\left( \frac{1-\varepsilon }{4},0,\frac{1+\varepsilon }{2},\frac{ 1-\varepsilon }{4}\right)$	0.5139	ESD	0.3124	ESD
$W_{10}=\left( \frac{1-\varepsilon }{4},\frac{1-\varepsilon }{4},0,\frac{ 1+\varepsilon }{2}\right)$	2.8819	ESB	0.4422	ESB
$W_{11}=\left( \frac{1+\varepsilon }{2},\frac{1-\varepsilon }{6},\frac{ 1-\varepsilon }{6},\frac{1-\varepsilon }{6}\right)$	0.7652	ESD	0.5929	ESB
$W_{12}=\left( \frac{1-\varepsilon }{6},\frac{1+\varepsilon }{2},\frac{ 1-\varepsilon }{6},\frac{1-\varepsilon }{6}\right)$	0.7652	ESD	0.6036	ESD
$W_{13}=\left( \frac{1-\varepsilon }{6},\frac{1-\varepsilon }{6},\frac{ 1+\varepsilon }{2},\frac{1-\varepsilon }{6}\right)$	0.5444	ESD	0.1996	ESD
$W_{14}=\left( \frac{1-\varepsilon }{6},\frac{1-\varepsilon }{6},\frac{ 1-\varepsilon }{6},\frac{1+\varepsilon }{2}\right)$	2.2990	ESB	0.4406	ESB

## Supplementary Information

Supplementary Information.

## Data Availability

All data generated or analysed during this study are included in this published article (and its Supplementary Information files). The codes used during the current study are available from the corresponding author on reasonable request.

## Code availability

The codes used during the current study are available from the corresponding author on reasonable request.

## References

[1] Życzkowski, K., Horodecki, P., Horodecki, M. & Horodecki, R. Dynamics of quantum entanglement. *Phys. Rev. A* **65**, 012101. 10.1103/PhysRevA.65.012101 (2001).

[2] Horodecki, R., Horodecki, P., Horodecki, M. & Horodecki, K. Quantum entanglement. *Rev. Mod. Phys.* **81**, 865–942. 10.1103/RevModPhys.81.865 (2009).

[3] Bennett, C. H. & DiVincenzo, D. P. Quantum information and computation. *Nature* **404**, 247–255 (2000). 10749200 10.1038/35005001

[4] Khalili, F. Y. & Polzik, E. S. Overcoming the standard quantum limit in gravitational wave detectors using spin systems with a negative effective mass. *Phys. Rev. Lett.* **121**, 031101. 10.1103/PhysRevLett.121.031101 (2018). 30085801 10.1103/PhysRevLett.121.031101

[5] Zeuthen, E., Polzik, E. S. & Khalili, F. Y. Gravitational wave detection beyond the standard quantum limit using a negative-mass spin system and virtual rigidity. *Phys. Rev. D* **100**, 062004 (2019).

[6] Ekert, A. K. Quantum cryptography based on bell’s theorem. *Phys. Rev. Lett.* **67**, 661–663. 10.1103/PhysRevLett.67.661 (1991). 10044956 10.1103/PhysRevLett.67.661

[7] Shor, P. W. & Preskill, J. Simple proof of security of the bb84 quantum key distribution protocol. *Phys. Rev. Lett.* **85**, 441 (2000). 10991303 10.1103/PhysRevLett.85.441

[8] Gisin, N., Ribordy, G., Tittel, W. & Zbinden, H. Quantum cryptography. *Rev. Mod. Phys.* **74**, 145 (2002).

[9] Bennett, C. H. Quantum information. *Phys. Scr.* **1998**, 210 (1998).

[10] DiVincenzo, D. P. Quantum computation. *Science* **270**, 255–261 (1995).

[11] Steane, A. Quantum computing. *Rep. Prog. Phys.* **61**, 117 (1998).

[12] DiVincenzo, D. P. The physical implementation of quantum computation. *Fortschritte der Physik: Progr. Phys.* **48**, 771–783 (2000).

[13] Ladd, T. D. et al. Quantum computers. *Nature* **464**, 45–53 (2010). 20203602 10.1038/nature08812

[14] Nielsen, M. A. & Chuang, I. L. *Quantum computation and quantum information* (Cambridge university press, 2010).

[15] Madsen, L. S. et al. Quantum computational advantage with a programmable photonic processor. *Nature* **606**, 75–81 (2022). 35650354 10.1038/s41586-022-04725-x PMC9159949

[16] Preskill, J. Quantum computing in the nisq era and beyond. *Quantum* **2**, 79 (2018).

[17] Riera-Sàbat, F., Sekatski, P. & Dür, W. Remotely controlled entanglement generation. *Quantum* **7**, 904 (2023).

[18] Zhang, Z. et al. Entanglement-based quantum information technology: a tutorial. *Adv. Opt. Photon.* **16**, 60–162 (2024).

[19] Wang, T.-L. et al. Remote entanglement generation via enhanced quantum state transfer. arXiv:2506.06669 (2025).

[20] Arute, F. et al. Quantum supremacy using a programmable superconducting processor. *Nature* **574**, 505–510 (2019). 31645734 10.1038/s41586-019-1666-5

[21] Cirac, J. I. & Zoller, P. Quantum computations with cold trapped ions. *Phys. Rev. Lett.* **74**, 4091–4094. 10.1103/PhysRevLett.74.4091 (1995). 10058410 10.1103/PhysRevLett.74.4091

[22] Monroe, C. et al. Large-scale modular quantum-computer architecture with atomic memory and photonic interconnects. *Phys. Rev. A* **89**, 022317 (2014).

[23] Zhong, H.-S. et al. Quantum computational advantage using photons. *Science* **370**, 1460–1463 (2020). 33273064 10.1126/science.abe8770

[24] Humphreys, P. C. et al. Deterministic delivery of remote entanglement on a quantum network. *Nature* **558**, 268–273 (2018). 29899475 10.1038/s41586-018-0200-5

[25] Tyryshkin, A. M. et al. Electron spin coherence exceeding seconds in high-purity silicon. *Nat. Mater.* **11**, 143–147 (2012). 10.1038/nmat3182 22138791

[26] Pla, J. J. et al. High-fidelity readout and control of a nuclear spin qubit in silicon. *Nature* **496**, 334–338 (2013). 23598342 10.1038/nature12011

[27] Watson, T. F. et al. A programmable two-qubit quantum processor in silicon. *Nature* **555**, 633–637 (2018). 29443962 10.1038/nature25766

[28] Loss, D. & DiVincenzo, D. P. Quantum computation with quantum dots. *Phys. Rev. A* **57**, 120–126. 10.1103/PhysRevA.57.120 (1998).

[29] Nowack, K. C., Koppens, F., Nazarov, Y. V. & Vandersypen, L. Coherent control of a single electron spin with electric fields. *Science* **318**, 1430–1433 (2007). 17975030 10.1126/science.1148092

[30] Petta, J. R. et al. Coherent manipulation of coupled electron spins in semiconductor quantum dots. *Science* **309**, 2180–2184 (2005). 16141370 10.1126/science.1116955

[31] Zajac, D. M. et al. Resonantly driven cnot gate for electron spins. *Science* **359**, 439–442 (2018). 29217586 10.1126/science.aao5965

[32] Bluhm, H. et al. Dephasing time of gaas electron-spin qubits coupled to a nuclear bath exceeding 200 s. *Nat. Phys.* **7**, 109–113 (2011).

[33] Chen, S.-H. Origin and early growth of entanglement by exchange with gate voltage controllable outcome. *Phys. Rev. B* **109**, 045308. 10.1103/PhysRevB.109.045308 (2024).

[34] Lin, L.-C., Tan, S. G., Chang, C.-R., Sun, S.-J. & Chen, S.-H. Entanglement induced by heisenberg exchange between an electron in a nested quantum dot and a qubit with relative motion. *New J. Phys.* (2025).

[35] Vavilov, M. G. & Glazman, L. I. Transport spectroscopy of kondo quantum dots coupled by rkky interaction. *Phys. Rev. Lett.* **94**, 086805. 10.1103/PhysRevLett.94.086805 (2005). 15783918 10.1103/PhysRevLett.94.086805

[36] Petersson, K. D. et al. Circuit quantum electrodynamics with a spin qubit. *Nature* **490**, 380–383 (2012). 23075988 10.1038/nature11559

[37] Yang, G., Hsu, C.-H., Stano, P., Klinovaja, J. & Loss, D. Long-distance entanglement of spin qubits via quantum hall edge states. *Phys. Rev. B* **93**, 075301. 10.1103/PhysRevB.93.075301 (2016).

[38] Wang, J.-N. et al. Unified formulations for rkky interaction, side kondo behavior, and fano antiresonance in a hybrid tripartite quantum dot device with filtered density of states. *Phys. Rev. B* **106**, 035428. 10.1103/PhysRevB.106.035428 (2022).

[39] Vonhoff, F., Fischer, A., Deltenre, K. & Anders, F. B. Microscopic origin of the effective spin-spin interaction in a semiconductor quantum dot ensemble. *Phys. Rev. Lett.* **129**, 167701. 10.1103/PhysRevLett.129.167701 (2022). 36306758 10.1103/PhysRevLett.129.167701

[40] Utsumi, Y., Martinek, J., Bruno, P. & Imamura, H. Indirect exchange interaction between two quantum dots in an aharonov-bohm ring. *Phys. Rev. B* **69**, 155320 (2004).

[41] Stocker, L. & Zilberberg, O. Coherent exchange-coupled nonlocal kondo impurities. *Phys. Rev. Res.* **6**, L022058 (2024).

[42] Chen, S.-H., Maekawa, S., Liu, M.-H. & Chang, C.-R. Mirror symmetry and exchange of magnetic impurities mediated by electrons of rashba spin-orbit interaction in a four-terminal landauer setup. *J. Phys. D Appl. Phys.* **43**, 015003 (2009).

[43] Allerdt, A., Büsser, C. A., Martins, G. B. & Feiguin, A. E. Kondo versus indirect exchange: Role of lattice and actual range of rkky interactions in real materials. *Phys. Rev. B* **91**, 085101. 10.1103/PhysRevB.91.085101 (2015).

[44] Mousavi, F. M. & Farghadan, R. Electrical control of ruderman-kittel-kasuya-yosida exchange interaction in zigzag edge mos2 nanoflakes. *J. Phys. Chem. Solids* **158**, 110242 (2021).

[45] Kettemann, S. Competition between kondo effect and rkky coupling. arXiv:2408.03112 (2024).

[46] Ruderman, M. A. & Kittel, C. Indirect exchange coupling of nuclear magnetic moments by conduction electrons. *Phys. Rev.* **96**, 99 (1954).

[47] Kasuya, T. A theory of metallic ferro- and antiferromagnetism. *Progr. Theoret. Phys.* **16**, 45 (1956).

[48] Yosida, K. Magnetic properties of cu-mn alloys. *Phys. Rev.* **106**, 893 (1957).

[49] Cho, S. Y. & McKenzie, R. H. Quantum entanglement in the two-impurity kondo model. *Phys. Rev. A* **73**, 012109. 10.1103/PhysRevA.73.012109 (2006).

[50] Elman, S. J., Bartlett, S. D. & Doherty, A. C. Long-range entanglement for spin qubits via quantum hall edge modes. *Phys. Rev. B* **96**, 115407. 10.1103/PhysRevB.96.115407 (2017).

[51] Klotz, M., Tangemann, A., Opferkuch, D. & Kubanek, A. Bipartite entanglement in a nuclear spin register mediated by a quasi-free electron spin. *Nat. Commun.* **17**, 2325. 10.1038/s41467-026-70154-3 (2026). 41792132 10.1038/s41467-026-70154-3 PMC12976098

[52] Yu, T. & Eberly, J. H. Finite-time disentanglement via spontaneous emission. *Phys. Rev. Lett.* **93**, 140404. 10.1103/PhysRevLett.93.140404 (2004). 15524773 10.1103/PhysRevLett.93.140404

[53] Yu, T. & Eberly, J. Sudden death of entanglement: Classical noise effects. *Opt. Commun.* **264**, 393–397 (2006).

[54] Yu, T. & Eberly, J. Quantum open system theory: Bipartite aspects. *Phys. Rev. Lett.* **97**, 140403 (2006). 17155224 10.1103/PhysRevLett.97.140403

[55] Ann, K. & Jaeger, G. Local-dephasing-induced entanglement sudden death in two-component finite-dimensional systems. *Phys. Rev. A* **76**, 044101. 10.1103/PhysRevA.76.044101 (2007).

[56] Yu, T. & Eberly, J. H. Sudden death of entanglement. *Science* **323**, 598–601 (2009). 19179521 10.1126/science.1167343

[57] Yuan, X.-Z., Goan, H.-S. & Zhu, K.-D. Non-Markovian reduced dynamics and entanglement evolution of two coupled spins in a quantum spin environment. *Phys. Rev. B* **75**, 045331. 10.1103/PhysRevB.75.045331 (2007).

[58] Wang, F. et al. Observation of entanglement sudden death and rebirth by controlling a solid-state spin bath. *Phys. Rev. B* **98**, 064306. 10.1103/PhysRevB.98.064306 (2018).

[59] Chen, S.-H., Tan, S. G. & Huang, C.-C. General recipe for immediate entanglement death and birth via bell states: environmental heisenberg exchange with transition as an example. *Phys. Scr.* **100**, 065114 (2025).

[60] Almeida, M. P. et al. Environment-induced sudden death of entanglement. *Science* **316**, 579–582 (2007). 17463284 10.1126/science.1139892

[61] Hutton, A. & Bose, S. Mediated entanglement and correlations in a star network of interacting spins. *Phys. Rev. A* **69**, 042312. 10.1103/PhysRevA.69.042312 (2004).

[62] Bazhanov, D. I., Sivkov, I. N. & Stepanyuk, V. S. Engineering of entanglement and spin state transfer via quantum chains of atomic spins at large separations. *Sci. Rep.* **8**, 14118 (2018). 30237521 10.1038/s41598-018-32145-3 PMC6148274

[63] Stocker, L., Sack, S. H., Ferguson, M. S. & Zilberberg, O. Entanglement-based observables for quantum impurities. *Phys. Rev. Res.* **4**, 043177. 10.1103/PhysRevResearch.4.043177 (2022).

[64] Mondal, P., Suresh, A. & Nikolić, B. K. When can localized spins interacting with conduction electrons in ferro- or antiferromagnets be described classically via the landau-lifshitz equation: Transition from quantum many-body entangled to quantum-classical nonequilibrium states. *Phys. Rev. B* **104**, 214401. 10.1103/PhysRevB.104.214401 (2021).

[65] Garcia-Gaitan, F. & Nikolić, B. K. Fate of entanglement in magnetism under Lindbladian or non-Markovian dynamics and conditions for their transition to Landau-Lifshitz-Gilbert classical dynamics. *Phys. Rev. B* **109**, L180408. 10.1103/PhysRevB.109.L180408 (2024).

[66] Mortezapour, A., Borji, M. A. & Franco, R. L. Protecting entanglement by adjusting the velocities of moving qubits inside non-markovian environments. *Laser Phys. Lett.* **14**, 055201 (2017).

[67] Huan, T., Zhou, R. & Ian, H. Dynamic entanglement transfer in a double-cavity optomechanical system. *Phys. Rev. A* **92**, 022301. 10.1103/PhysRevA.92.022301 (2015).

[68] Obada, A. F., Hessian, H. & Hashem, M. Quantum entanglement in a system of two moving atoms interacting with a single mode field. *Phys. Scr.* **81**, 055303 (2010).

[69] Pandit, M., Das, S., Roy, S. S., Dhar, H. S. & Sen, U. Effects of cavity-cavity interaction on the entanglement dynamics of a generalized double jaynes-cummings model. *J. Phys. B: At. Mol. Opt. Phys.* **51**, 045501 (2018).

[70] Costa, A. Jr. & Bose, S. Impurity scattering induced entanglement of ballistic electrons. *Phys. Rev. Lett.* **87**, 277901 (2001). 11800916 10.1103/PhysRevLett.87.277901

[71] Sharma, A. & Tulapurkar, A. A. Transmission-based tomography for spin qubits. *Phys. Rev. A* **103**, 052430 (2021).

[72] Leon, A. O., d’Albuquerque e Castro, J., Retamal, J. C., Cahaya, A. B. & Altbir, D.,. Manipulation of the rkky exchange by voltages. *Phys. Rev. B* **100**, 014403. 10.1103/PhysRevB.100.014403 (2019).

[73] Tran, B. X. et al. Field-free control and switching of perpendicular magnetization by voltage induced manipulation of rkky interaction. *Appl. Phys. Lett.* **124** (2024).

[74] Trényi, R. et al. Activation of metrologically useful genuine multipartite entanglement. *New J. Phys.* **26**, 023034 (2024).

[75] Hahn, E. L. Spin echoes. *Phys. Rev.* **80**, 580–594. 10.1103/PhysRev.80.580 (1950).

[76] Yosida, K. Bound state due to the exchange interaction. *Phys. Rev.* **147**, 223–227. 10.1103/PhysRev.147.223 (1966).

[77] Allerdt, A., Feiguin, A. E. & Das Sarma, S. Competition between kondo effect and rkky physics in graphene magnetism. *Phys. Rev. B* **95**, 104402 (2017). 10.1103/PhysRevB.95.104402.

[78] Doniach, S. The kondo lattice and weak antiferromagnetism. *Physica B+C* **91**, 231–234 (1977). https://www.sciencedirect.com/science/article/pii/0378436377901905.

[79] Kroha, J. Interplay of Kondo effect and RKKY interaction. In Pavarini, E., Koch, E., Scalettar, R. & Martin, R. M. (eds.) *The Physics of Correlated Insulators, Metals, and Superconductors Modeling and Simulation*, vol. 7 (Verlag des Forschungszentrum Jülich, 2017).

[80] Hill, S. A. & Wootters, W. K. Entanglement of a pair of quantum bits. *Phys. Rev. Lett.* **78**, 5022–5025. 10.1103/PhysRevLett.78.5022 (1997).

[81] Wootters, W. K. Entanglement of formation of an arbitrary state of two qubits. *Phys. Rev. Lett.* **80**, 2245–2248. 10.1103/PhysRevLett.80.2245 (1998).

[82] Rungta, P., Bužek, V., Caves, C. M., Hillery, M. & Milburn, G. J. Universal state inversion and concurrence in arbitrary dimensions. *Phys. Rev. A* **64**, 042315. 10.1103/PhysRevA.64.042315 (2001).

[83] Walls, D. F. Squeezed states of light. *Nature* **306**, 141–146 (1983).

[84] Wu, L.-A., Xiao, M. & Kimble, H. Squeezed states of light from an optical parametric oscillator. *J. Opt. Soc. Am. B* **4**, 1465–1475 (1987).

[85] Pirkkalainen, J.-M., Damskägg, E., Brandt, M., Massel, F. & Sillanpää, M. A. Squeezing of quantum noise of motion in a micromechanical resonator. *Phys. Rev. Lett.* **115**, 243601. 10.1103/PhysRevLett.115.243601 (2015). 26705631 10.1103/PhysRevLett.115.243601

[86] Marti, S. et al. Quantum squeezing in a nonlinear mechanical oscillator. *Nat. Phys.* **20**, 1448–1453 (2024).

