# Supplementary material for: Navigating entanglement via Ruderman–Kittel–Kasuya–Yosida exchange: oscillatory, boundary-residing, pulsed, and damping-stabilized trajectories
Source: Sci Rep. 2026 Apr 10;16:16942. doi: 10.1038/s41598-026-47292-1 (PMC13230907; doi:10.1038/s41598-026-47292-1)
Supplement: Supplementary file 1 — Supplementary Information. [file 41598_2026_47292_MOESM1_ESM.pdf]

Supplementary Information for: Navigating entanglement via Ruderman-Kittel-Kasuya-Yosida exchange: Oscillatory, boundary-residing, pulsed, and damping-stabilized trajectories

Son-Hsien Chen *

Department of Applied Physics and Chemistry, University of Taipei, Taipei 100234, Taiwan

Seng Ghee Tan

Department of Optoelectric Physics, Chinese Culture University, Taipei 11114, Taiwan

Ching-Ray Chang

Quantum Information Center, Chung Yuan Christian University, Taoyuan, 320314, Taiwan

S1: EXTENDED CONCURRENCE IN TERMS OF ETI FOR MIXED STATES AND EXISTENCE OF SNAKE TRAJECTORY

In contrast to pure states, for mixed states of the form (27), the evolution of the reduced DM $\rho(t)$ remains block diagonal. In this appendix, we demonstrate that such a structure allows the extended concurrence $\mathcal{C}_E(t)$ to be explicitly expressed as a functional of the ETI $I(t)$, providing a direct and compact method to track the entanglement evolution. In particular, we show that snake trajectories can be identified analytically without invoking the ESP ε.

Consider the spin-up IS with $S^C = \hbar/2$ and the qubits are described by (27). We set $\gamma = 1$ for isotropic exchange. Using (19) and (21) and defining the weight sums and differences as $w_s = w_{\alpha^+} + w_{\alpha^-}$ and $w_d = |w_{\alpha^+} - w_{\alpha^-}| > 0$, we introduce the functional dependence on the ETI via

$$C(I) = \cos(3I), \tag{S1.1}$$

and

$$k(I) = 1 - C(I) \in [0, 2], \tag{S1.2}$$

* sonhsien@utaipei.edu.tw

the reduced DM $\rho(t)$ then takes the form

$$\begin{pmatrix} \frac{9w_s+8w_{\beta+}k}{18} & 0 & 0 & \frac{w_d}{6}\left(1+2e^{-3iI}\right) \\ 0 & \frac{X^+}{18} & \frac{X^-}{18} & 0 \\ 0 & \frac{X^-}{18} & \frac{X^+}{18} & 0 \\ \frac{w_d}{6}\left(1+2e^{3iI}\right) & 0 & 0 & \frac{w_s(9-4k)}{18} \end{pmatrix}, \tag{S1.3}$$

where $X^{\pm}=X\pm 9w_{\beta-}$ and

$$X=9w_{\beta+}+2k\left(w_s-2w_{\beta+}\right). \tag{S1.4}$$

Recall that $\mathcal{C}_E(t)$ is constructed from the eigenvalues of (23), i.e., the square root of the eigenvalues ($\sqrt{\widetilde{\kappa}_i}=\kappa_i$, $i\in\{1,2,3,4\}$) of

$$\sqrt{\rho(t)}\rho'(t)\sqrt{\rho(t)}. \tag{S1.5}$$

Since $\rho(t)$ and $\rho'(t)$ are positive semi-definite Hermitian matrices, Eq. (S1.5) and

$$R(t)=\rho(t)\rho'(t) \tag{S1.6}$$

share the same eigenvalues $\widetilde{\kappa}_i$. Substituting (S1.3) into this expression, we obtain $R(t)$ as

$$\begin{pmatrix} \frac{9-4k}{162}\left(\frac{9}{2}(w_s^2+w_d^2)+4w_sw_{\beta+}k\right) & 0 & 0 & \frac{w_d}{54}(1+2e^{-3iI})(9w_s+8w_{\beta+}k) \\ 0 & \frac{X^2+81w_{\beta-}^2}{162} & \frac{X^2-81w_{\beta-}^2}{162} & 0 \\ 0 & \frac{X^2-81w_{\beta-}^2}{162} & \frac{X^2+81w_{\beta-}^2}{162} & 0 \\ \frac{w_sw_d}{54}(1+2e^{3iI})(9-4k) & 0 & 0 & \frac{9-4k}{162}\left(\frac{9}{2}(w_s^2+w_d^2)+4w_sw_{\beta+}k\right) \end{pmatrix}. \tag{S1.7}$$

Solving for the square roots of the eigenvalues of $R(t)$ yields

$$\kappa_1(k)=\frac{9w_{\beta+}+2(w_s-2w_{\beta+})k}{9} \tag{S1.8}$$

$$\kappa_2(k)=\frac{\sqrt{9-4k}}{18}\left(\sqrt{w_s(9w_s+8w_{\beta+}k)}-3w_d\right) \tag{S1.9}$$

$$\kappa_3(k) = \frac{\sqrt{9-4k}}{18}\left(\sqrt{w_s(9w_s + 8w_{\beta^+}k)} + 3w_d\right) \tag{S1.10}$$

and

$$\kappa_4 = w_{\beta^-}. \tag{S1.11}$$

Note that $\kappa_3(k) > \kappa_2(k)$ as seen from the sign of the last term; thus, the maximum eigenvalue must be $\kappa_{\max} \in \{\kappa_1, \kappa_3, \kappa_4\}$.

As k increases, the condition

$$w_s > 2w_{\beta^+} \tag{S1.12}$$

ensures that $\kappa_1(k)$ is an increasing function of k. Conversely, $\kappa_3(k)$ decreases with k because the decay of the prefactor $\sqrt{9-4k}$ dominates the growth of $\sqrt{w_s(9w_s + 8w_{\beta^+}k)}$. The equality $\kappa_1(k) = \kappa_3(k)$ always occurs within the interval $k \in [0, 2]$. This can be verified by analyzing the boundary values:

$$\kappa_1(0) = w_{\beta^+} \tag{S1.13}$$

$$\kappa_3(0) = \frac{w_s + w_d}{2} \tag{S1.14}$$

$$\kappa_1(2) = \frac{4w_s + w_{\beta^+}}{9} \tag{S1.15}$$

$$\kappa_3(2) = \frac{\sqrt{w_s(9w_s + 16w_{\beta^+})} + 3w_d}{18}. \tag{S1.16}$$

Under the condition in Eq. (S1.12), these boundaries imply $\kappa_1(0) - \kappa_3(0) < 0$ and $\kappa_1(2) - \kappa_3(2) > 0$. For simplicity, if we assume

$$w_{\beta^-} = 0, \tag{S1.17}$$

then $\kappa_4 = 0$ and thus $\kappa_{\max} \in \{\kappa_1, \kappa_3\}$. Under this assumption, at the crossing point where $\kappa_1 = \kappa_3$, Eq. (22) guarantees that $\mathcal{C}_E = -\kappa_2 < 0$. Consequently, the question of whether a snake trajectory exists reduces to determining if a positive $\mathcal{C}_E > 0$ exists elsewhere within $k \in [0, 2]$. In fact, under the two conditions (S1.12) and (S1.17), we always find a positive value for $\mathcal{C}_E$ at $k = 2$, $\mathcal{C}_E|_{k=2} = \kappa_1(2) - \kappa_2(2) - \kappa_3(2) > 0$, since $\kappa_1(2) > \kappa_2(2) + \kappa_3(2) = \sqrt{w_s(9w_s + 16w_{\beta^+})}/9$. Therefore, these two conditions—while not optimized for maximizing the balance between the entangled and separable residence times—are sufficient to produce a sign change in $\mathcal{C}_E$, signifying the emergence of the snake trajectory.

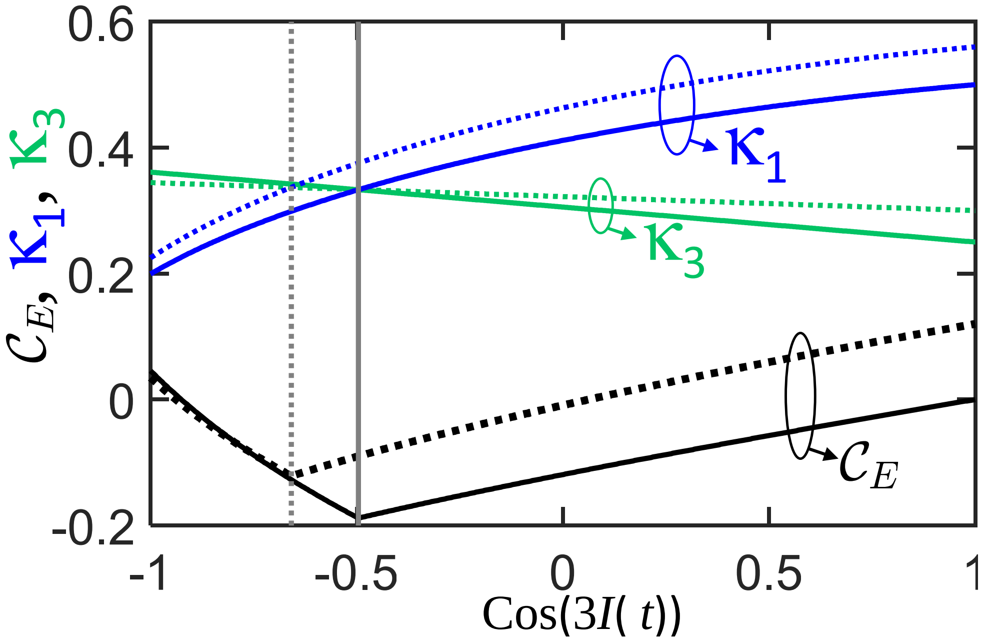

FIG. S1: Eigenvalues κ_1 (blue) and κ_3 (green), and extended concurrence $\mathcal{C}_E\,(t)$ (black) plotted as functions of $\cos\left(3I\left(t\right)\right)$. The solid lines correspond to the weighting $(w_{\alpha^+}, w_{\alpha^-}, w_{\beta^+}, w_{\beta^-}) = (0.5, 0.25, 0.25, 0)$, while the dotted lines use $(w_{\alpha^+}, w_{\alpha^-}, w_{\beta^+}, w_{\beta^-}) = (0.56, 0.14, 0.3, 0)$. Both weightings satisfy the conditions in Eqs. (S1.12) and (S1.17), which produce the snake trajectory. The vertical lines mark the points where κ_1 and κ_3 cross.

The above analysis is illustrated in Fig. S1, which plots κ_1, κ_3, and $\mathcal{C}_E$ as functions of $\cos\left(3I\left(t\right)\right)$. Under the conditions (S1.12) and (S1.17), a crossing of the eigenvalues κ_1 and κ_3 results in negative concurrence $\mathcal{C}_E < 0$, whereas positive concurrence $\mathcal{C}_E > 0$ is recovered at $\cos\left(3I\left(t\right)\right) = -1$ (corresponding to $k = 2$), thereby realizing the snake trajectory. By controlling the time spent in the positive and negative $\mathcal{C}_E$ regions via qubit motion, a programmable entanglement trajectory can be achieved.

S2: ANALYTIC ESTIMATION AND NUMERICAL VALIDATION OF THE CHARACTERISTIC PERIOD

For mixed states, the characteristic period T^*, defined by the separability-boundary criterion $\mathcal{C}_E(T^*/4) = 0$, can be estimated analytically under the short-time approximation. Based on the results in Table 2 of Physica Scripta 100, 065114 (2025), which assumes a constant exchange interaction $J^{A/B}(t) = J^{A/B}$, we compute the analytic expressions for T^* valid up to order $O(dt^2)$. These approximate values are listed in Table S2. To validate this approximation for our alternating-RKKY-exchange scheme, we compare the ETI calculated using these constant-exchange analytic periods against the full numerical results for sinusoidal exchange (as adopted in the main text). As shown in the last two columns of Table S2, the constant-exchange ETI is in good agreement with the exact numerical values, confirming that the short-time analytic model provides a reliable estimate for the near-boundary dynamics studied herein.

TABLE S2: Approximate analytic characteristic period T^* as a function of the entanglement switch parameter ε for *mixed* states, obtained with *time-independent* (constant) $J_x^{A/B}(t) = J_y^{AB}(t) = J_z^{AB}(t) = J_0$. Equivalent weightings that yield the same analytic form of T^* are grouped together. The expressions are valid up to order $O(dt^2)$, with dt the elapsed time after $t = 0$. The last two columns show the ETI for constant J^{AB} compared with the ETI for sinusoidal exchange in Table 1, calculated without the short-time approximation.

Weightings	Approximate T^* $(\hbar/J_0)$	Constant J, Approximate $I\left(\frac{T^*}{4}\right)$	Sinusoidal J, $I\left(\frac{T^*}{4}\right)$ (Table 1)
W_1, W_3, W_5	$\sqrt{\frac{16\varepsilon}{1+\varepsilon}}$	0.157	0.100
W_2, W_4	$2\sqrt{2}$	0.655	0.417
W_7, W_8	$\sqrt{\frac{32\varepsilon}{1+3\varepsilon}}$	0.220	0.140
W_9	$\sqrt{\frac{32\varepsilon}{3+5\varepsilon}}$	0.129	0.082
W_{10}	$\left[-\frac{1024\varepsilon(1+\varepsilon)}{(1-\varepsilon)^2}\right]^{1/4}$	0.721	0.459
W_{11}, W_{12}	$\sqrt{\frac{24\varepsilon}{(1+2\varepsilon)}}$	0.191	0.122
W_{13}	$\sqrt{\frac{12\varepsilon}{(1+2\varepsilon)}}$	0.136	0.087
W_{14}	$\left[-\frac{(384+768\varepsilon)\varepsilon}{(1-\varepsilon)^2}\right]^{1/4}$	0.575	0.366
